# Supplementary material for: Structural mechanism of BRD4-NUT and p300 bipartite interaction in propagating aberrant gene transcription in chromatin in NUT carcinoma
Source: Nat Commun. 2023 Jan 24;14:378. doi: 10.1038/s41467-023-36063-5 (PMC9870903; doi:10.1038/s41467-023-36063-5)
Supplement: Supplementary file 1 — Supplementary Information [file 41467_2023_36063_MOESM1_ESM.pdf]

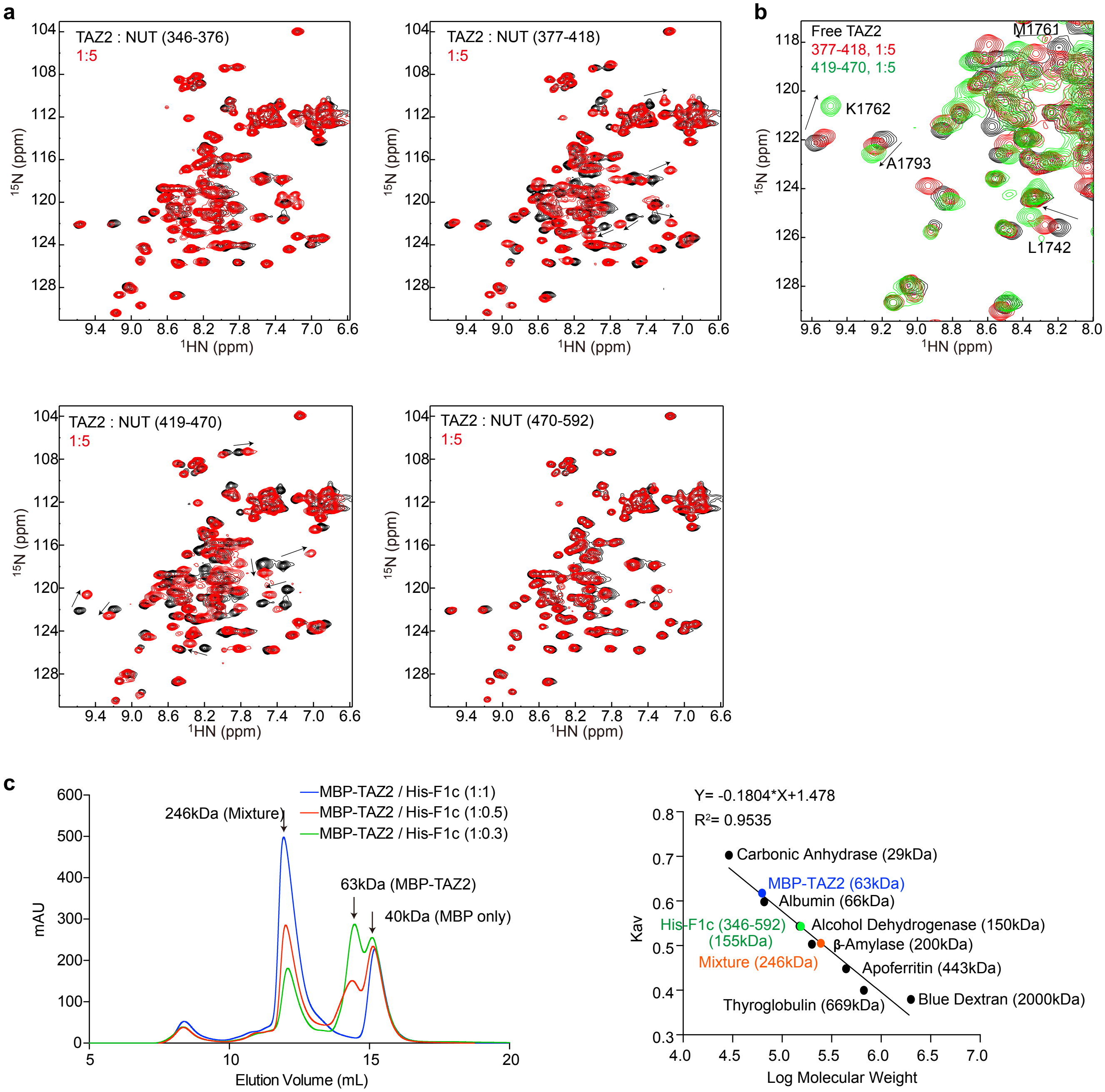

Supplementary Fig. 1 | NUT F1c region contains two TADs that bind to p300 TAZ2 domain.
(a), Superimposed ^{1}H,^{15}N-HSQC full spectra of ^{15}N-labeled p300 TAZ2 domain collected upon addition of different NUT-F1c protein fragments (red vs black). Peptides comprising NUT residues 346-376 and 470-592 did not induce TAZ2 chemical shift perturbation, indicating no binding, whereas peptides containing NUT residues 377-418 and 419-470 caused TAZ2 chemical shift perturbation, confirming their binding to the protein. **b**, Superimposed ^{1}H,^{15}N-HSQC spectra of ^{15}N-TAZ2 domain with two NUT-F1c long peptides as indicated. The NUT 419-470 sequence (green) induced similar but more dispersed resonance changes than the 377-418 sequence(red) when bound to the TAZ2 domain. **c**, Size-exclusion chromatography of p300 TAZ2/NUT F1c domain complexes. *Left* panel: Elution curves of protein complexes in indicated molar ratios; *Right* panel: Standard curve generated from a linear fit of the log molecular weight of the reference proteins versus their elution parameter Kav, where Kav=(Ve-Vo)/(Vcol-Vo). The MWs of MBP-TAZ2, His-F1c (346-592) and the protein complex were estimated on a linear fit to the molecular weight of reference proteins.

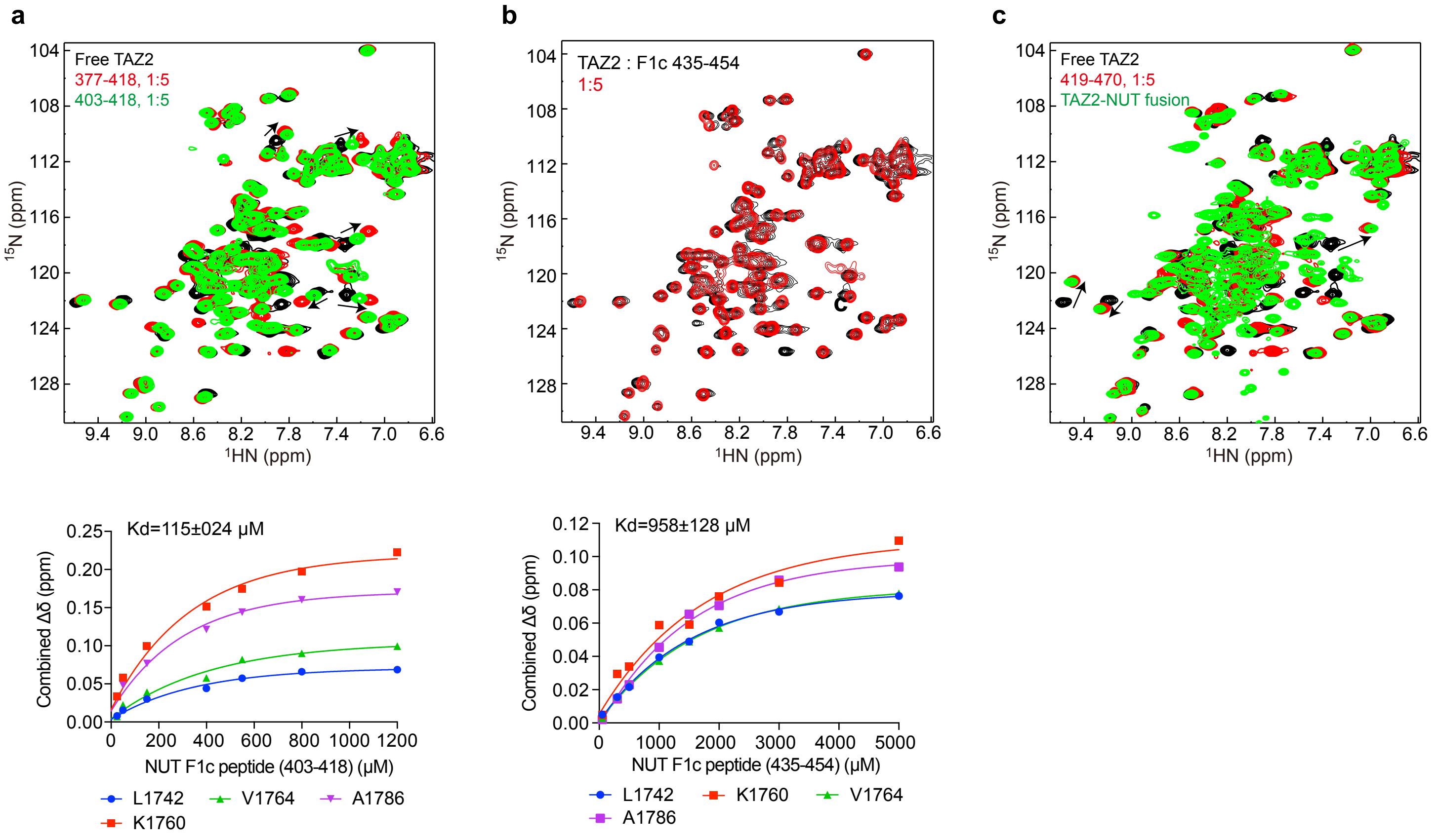

Supplementary Fig. 2 | Mapping of NUT F1c TAD motifs that bind to p300 TAZ2 domain.
a, Comparison of ^{15}N-HSQC expanded spectra of ^{15}N-labeled p300 TAZ2 domain titrated with NUT-F1c sequence (377-418, red) to (403-418, green) at indicated molar ratios. *Upper* panel, the NMR HSQC binding analysis of TAZ2 domain with short F1c peptides as indicated. The saturation curves were calculated by using a single-site binding equilibrium model. **b**, Superimposed ^{15}N-HSQC full spectra of ^{15}N-labeled p300 TAZ2 domain titrated F1c peptide (435-454, red). *Upper* panel, the F1c peptide binding affinity to p300 TAZ2 domain was determined by NMR titration as in **a**. **c**, Comparison of ^{15}N-HSQC TAZ2 spectrum with NUT-F1c long peptide (419-470) to that of TAZ2-NUT fusion protein containing the NUT 419-470 sequence.

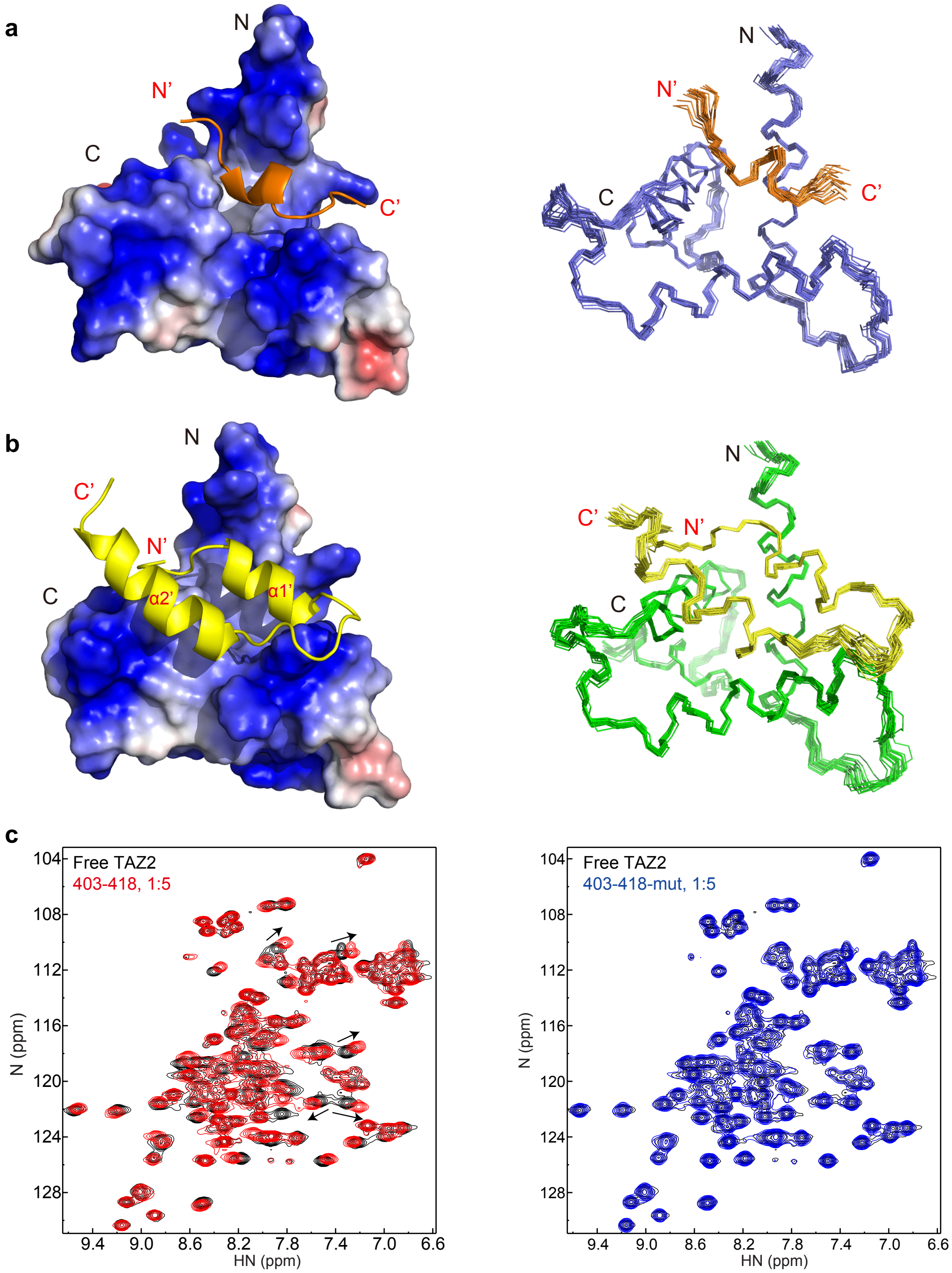

Supplementary Fig. 3 | Structures of p300 TAZ2 domain in complex with two different NUT F1c sequences. **a,** NMR structure of the p300 TAZ2 domain in complex with F1c peptide (aa 403-418). *Left* panel: Electrostatic potential surface representation; *Right*: The backboneatom superposition of ensembles of 20 NMR-derived structures of the complex; The electrostatic potential calculation was performed in PyMol (v 1.8). **b,** NMR structure of TAZ2-NUT fusion protein containing the F1c binding sequence (aa 419-470). *Left* panel: Electrostatic potential surface representation; *Right*: The backbone atom superposition of ensembles of 20 NMR-derived structures of the lowest energies; The electrostatic potential calculation was performed in PyMol (v 1.8). The TAZ2 is colored in green, and the NUT F1c sequence is colored in yellow. **c**, Comparison of ^{15}N-HSQC expanded spectra of ^{15}N-labeled p300 TAZ2 domain titrated with F1c peptide (403-418, red) (left) or the mutant peptide (403-418-mut, blue) (right) at indicated molar ratios.

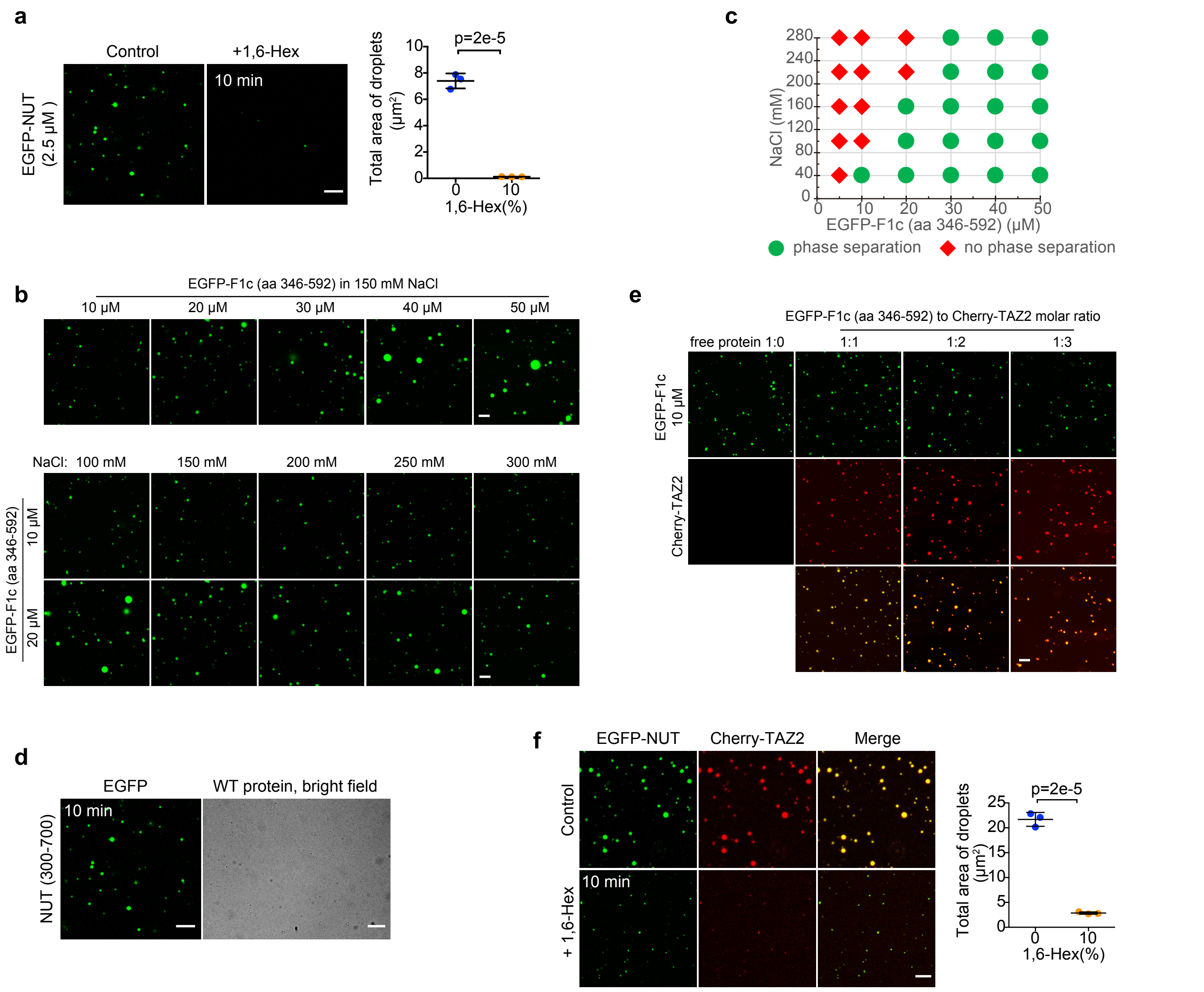

Supplementary Fig. 4 | NUT LLPS is facilitated by its p300 TAZ2 binding. a, LLPS droplets of EGFP-NUT (aa 300-700) (2.5 μM) are sensitive to 1,6-hexanediol (10%). Histogram plots showing decreases of total droplet areas per field (36 × 36 μm) in the absence/presence of 1,6-hexanediol. Three fields are quantified. Scalar bars: 5 μm. Quantitative data are shown as mean ± S.D. (n=minimum three independent experiments). Two-tailed unpaired Student's t-test without adjustment for multiple comparisons. **b**, Fluorescence images showing LLPS droplets of EGFP-F1c (aa 346-592) with increasing amounts of the protein (upper panel) or salt concentrations (lower panel) as indicated. Scalar bars: 5 μm. Experiments were repeated at least three times. **c**, Phase diagrams showing EGFP-F1c (aa 346-592) as a function of salt (NaCl) and protein concentrations. The green circles highlight phase separation and the red diamonds indicate no phase separation. **d**, Liquid droplet images of EGFP-tagged NUT (300-700) proteins in comparison to wild type proteins. Protein concentrations: EGFP-NUT 2.5 μM, NUT 25 μM. Scalar bars: EGFP-NUT 5 μm, NUT 50 μm. Experiments were repeated at least three times. **e**, Fluorescence images showing LLPS droplets of EGFP-F1c (aa 346-592, 10 μM) with addition of Cherry-TAZ2 as indicated molar ratios. Scalar bars: 5 μm. Experiments were repeated at least three times. **f**, Fluorescence images showing disruption of LLPS droplets of EGFP-NUT (aa 300-700, 2.5 μM) bound to Cherry-TAZ2 (1:3 molar ratio) upon addition of 10% 1,6-hexanediol (left panel). Histogram plots showing decreases of droplet areas per field (36 × 36 μm) in the presence/absence of 1,6-hexanediol. Three fields are quantified. Scalar bars: 5 μm. Quantitative data are shown as mean ± S.D. (n=minimum three independent experiments). Two tailed unpaired Student's t-test without adjustment for multiple comparisons.

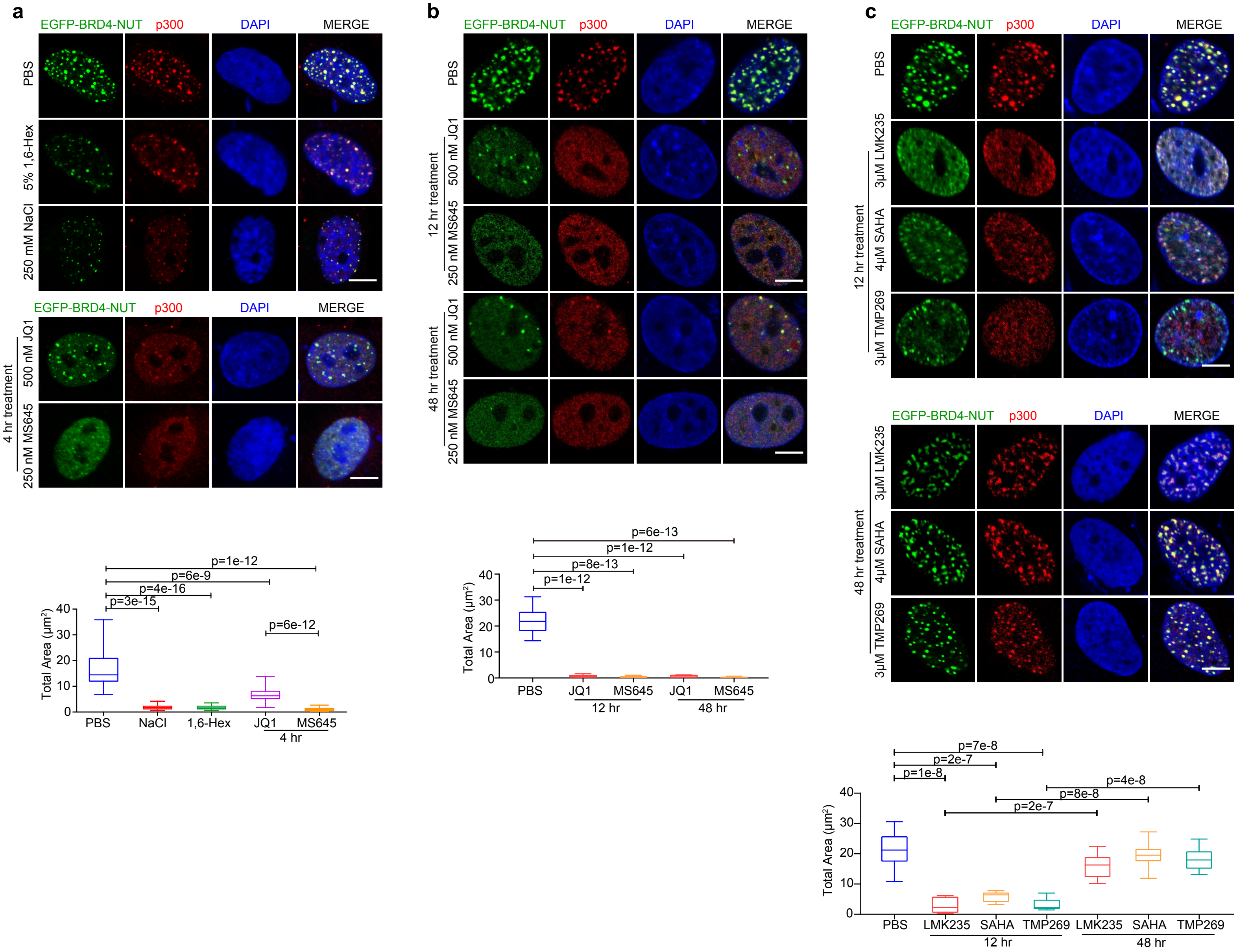

Supplementary Fig. 5 | Co-condensation of BRD4-NUT and p300 is dependent on TADs and sensitive to chemical treatment.
a, Immunofluorescence images of EGFP-BRD4-NUT-WT/p300 puncta in LO2 cell nuclei dispersed following the treatment of 5% 1,6-hexanediol, or NaCl (250 mM), BrD inhibitors JQ1 (500 nM) or MS645 (250 nM) (4 hours). Endogenous p300 was visualized using the p300 antibody. *Lower panel*, statistical analysis depict the total area of puncta per cell with treatment of 1,6-hexanediol, NaCl, JQ1 or MS645 measured for each sample using ImageJ. Box plots represent the lowest, lower quartile, median, upper quartile, and highest observations of puncta areas in cells with indicated treatments (n = minimum of 40 cells). Two-tailed unpaired Student's t-test without adjustment for multiple comparisons. Scalar bars: 5 μm. **b**, Immunofluorescence images of EGFP-BRD4-NUT-WT/p300 puncta in LO2 cell nuclei dispersed following treatment of BrD inhibitors JQ1 (500 nM) or MS645 (250 nM) for 12 or 48 hours. Endogenous p300 was visualized using the p300 antibody. *Lower panel*, statistical analysis depict the total area of puncta per cell with treatment of JQ1 and MS645 measured for each sample using ImageJ. Box plots represent the lowest, lower quartile, median, upper quartile, and highest observations of puncta areas in cells with indicated BETi treatments (n = minimum of 9 cells). Two-tailed unpaired Student's t-test without adjustment for multiple comparisons. Scalar bars: 5 μm. **c**, Immunofluorescence images showing EGFP-BRD4-NUT-WT puncta in colocalization with p300 in LO2 cells. The cells were treated with or without (PBS control) HDAC inhibitors LMK235 (3 μM), SAHA (4 μM) and TMP269 (3 μM) for 12 hours (top) or 48 hours (bottom) examined by immunofluorescence images. Endogenous p300 was visualized using the p300 antibody. *Lower panel*, statistical quantifications indicate the total area of puncta per cell with HDACi treatment measured for each sample using ImageJ. Box plots represent the lowest, lower quartile, median, upper quartile, and highest observations of puncta areas in cells with HDACi treatments (n = minimum of 9 cells). Two-tailed unpaired Student's t-test without adjustment for multiple comparisons. Scalar bars: 5 μm.

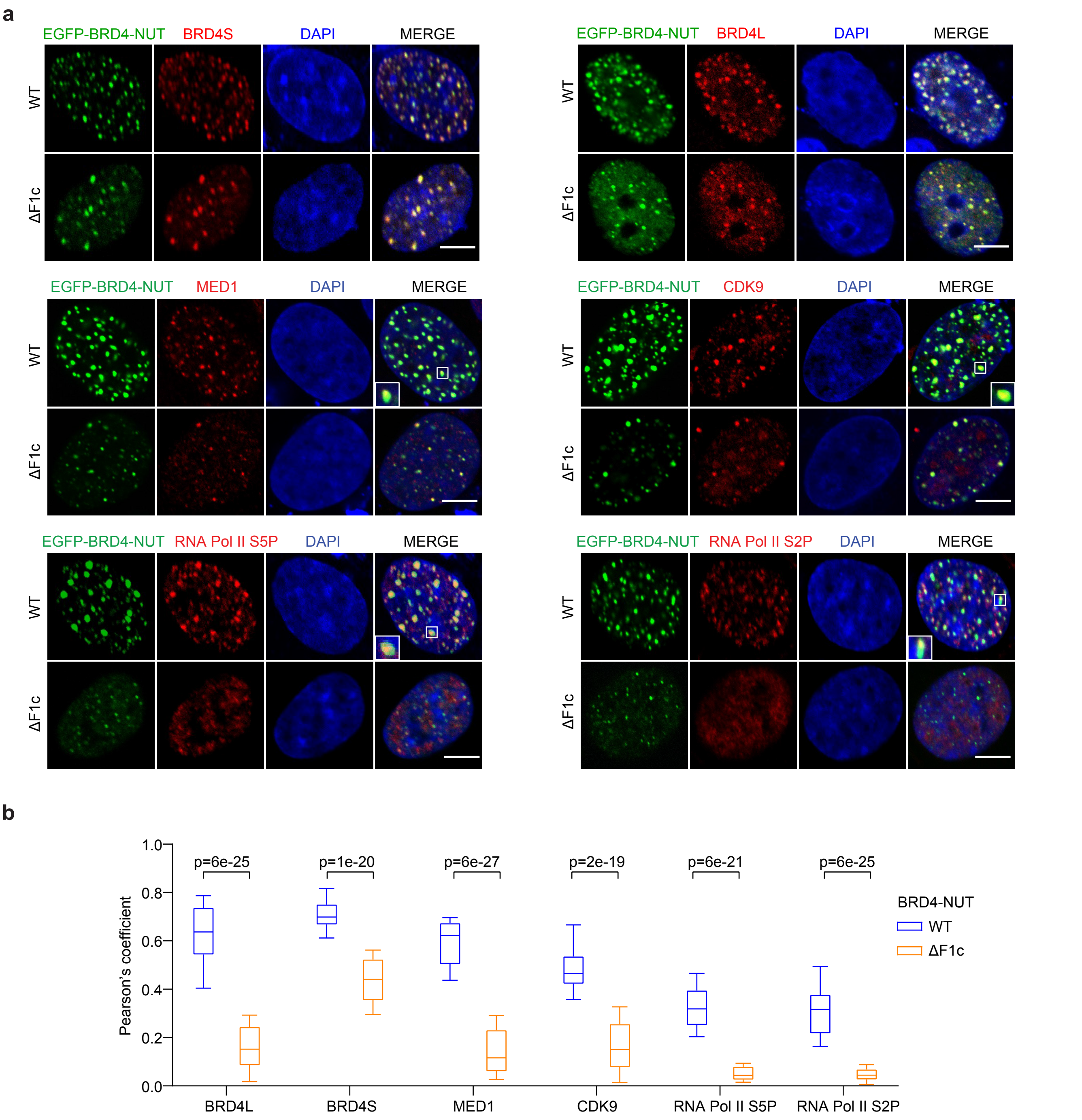

Supplementary Fig. 6 | BRD4-NUT co-condenation with general transcription machinery and cofactors in cells.
a, Microscopic images showing EGFP-BRD4-NUT-WT or EGFP-BRD4-NUT-ΔF1c puncta in partial cocondensation with general transcription machinery and general cofactors, FLAG-BRD4L, FLAG-BRD4S, MED1, CDK9 and RNA Pol II (S5P and S2P) in LO2 cell nuclei. Endogenous MED1, CDK9, RNA Pol II S5P and S2P were visualized using the FLAG-tag, MED1, CDK9, S5P and S2P antibodies. Scalar bars: 5 μm (n= minimum three independent experiments). **b**, Quantitative analysis of EGFR-BRD4-NUT-WT or EGFP-BRD4-NUT-ΔF1c co-localized with different trascription components. Pearson's correlation coefficients were for BRD4-NUT/BRD4L (WT: 0.63, ΔF1c: 0.16), BRD4-NUT/BRD4S (WT: 0.71, ΔF1c: 0.44), BRD4-NUT/MED1 (WT: 0.59, ΔF1c: 0.14), BRD4-NUT/CDK9 (WT: 0.48, ΔF1c: 0.16), BRD4-NUT/S5P (WT: 0.33, ΔF1c: 0.05), and BRD4-NUT/S2P (WT: 0.30, ΔF1c: 0.05). The coefficients were generated with three independent experiments by ImageJ, each with at least 30 cell images/group. Two-tailed unpaired Student's t-test without adjustment for multiple comparisons.

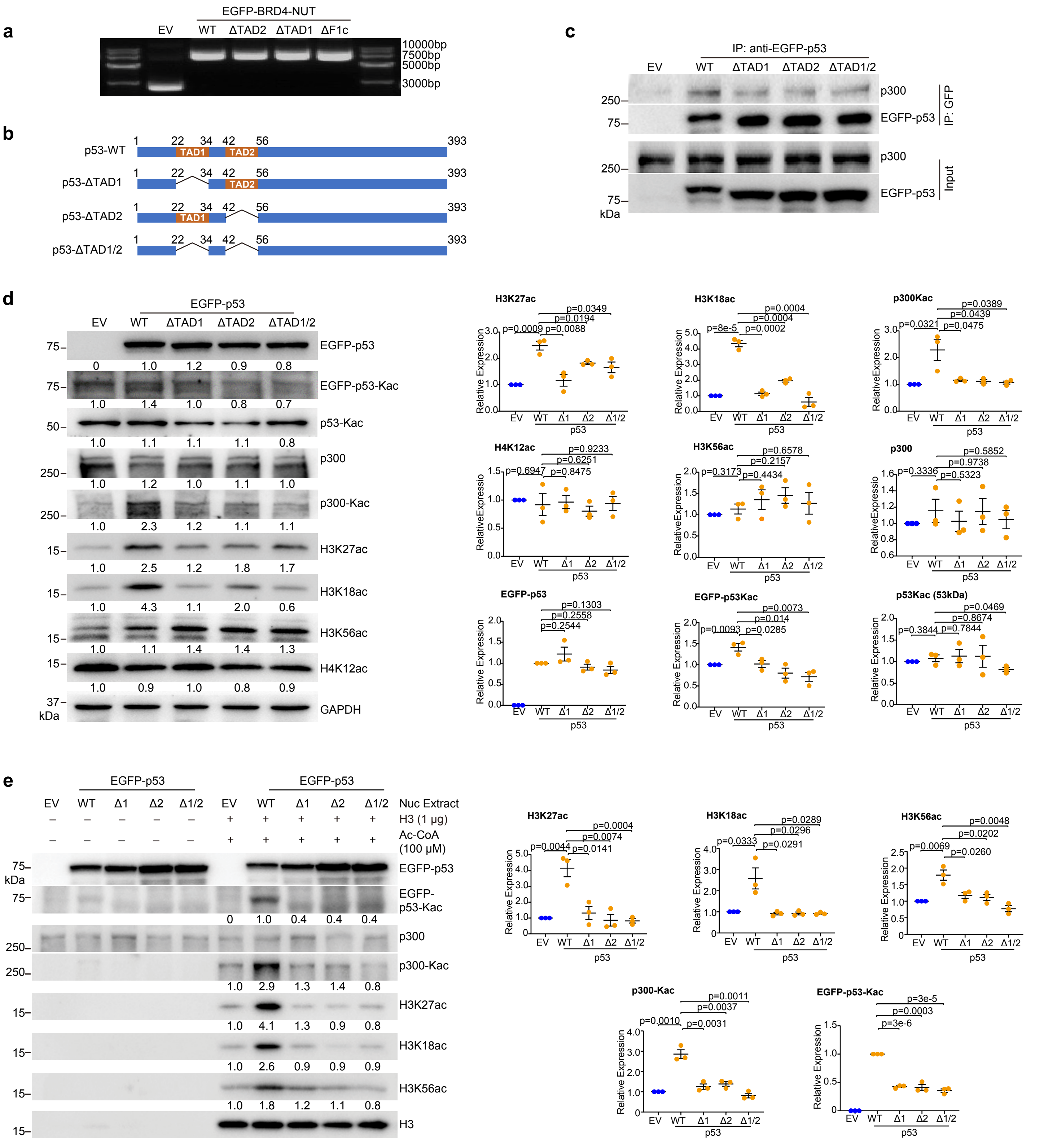

Supplementary Fig. 7 | p53 bipartite interaction with p300 induces p300 activation, p53 and histone acetylation.

a. Gel mobility shift assay with EV (Empty Vector), BRD4-NUT WT and deletion constructs. **b.** Schematic representations showing domain architecture of p53-WT or p53-ΔTAD1, -ΔTAD2 and -ΔTAD1/2. **c.** Co-IP experiments were performed in HEK293 cells transfected with EV (Empty Vector), p53-WT or its TAD deletion variants. Cell lysates were immunoprecipitated with anti-GFP antibody, and endogenous p300 was detected by anti-p300 antibody. (n=minimum three independent experiments). **d.** Cell lysates were analyzed by western blot using indicated antibodies. Quantitative data are shown as mean ± SEM (n=minimum three independent experiments). Two-tailed unpaired Student's t-test without adjustment for multiple comparisons. The relative expression was normalized by GAPDH. **e.** HEK293 cells were transfected with EV, p53-WT or the TAD deletion mutants. Equal amounts of nuclear extracts were analyzed for HAT activity by adding acetyl-CoA (100 μM) and recombinant H3 (1 μg) and visualized by indicated antibodies. Quantitative data are shown as mean ± SEM (n=minimum three independent experiments). Two-tailed unpaired Student's t-test without adjustment for multiple comparisons. The relative expressionof H3K18ac or H3K27ac was normalized by H3, p300-Kac by p300 and p53-Kac by p53, respectively.

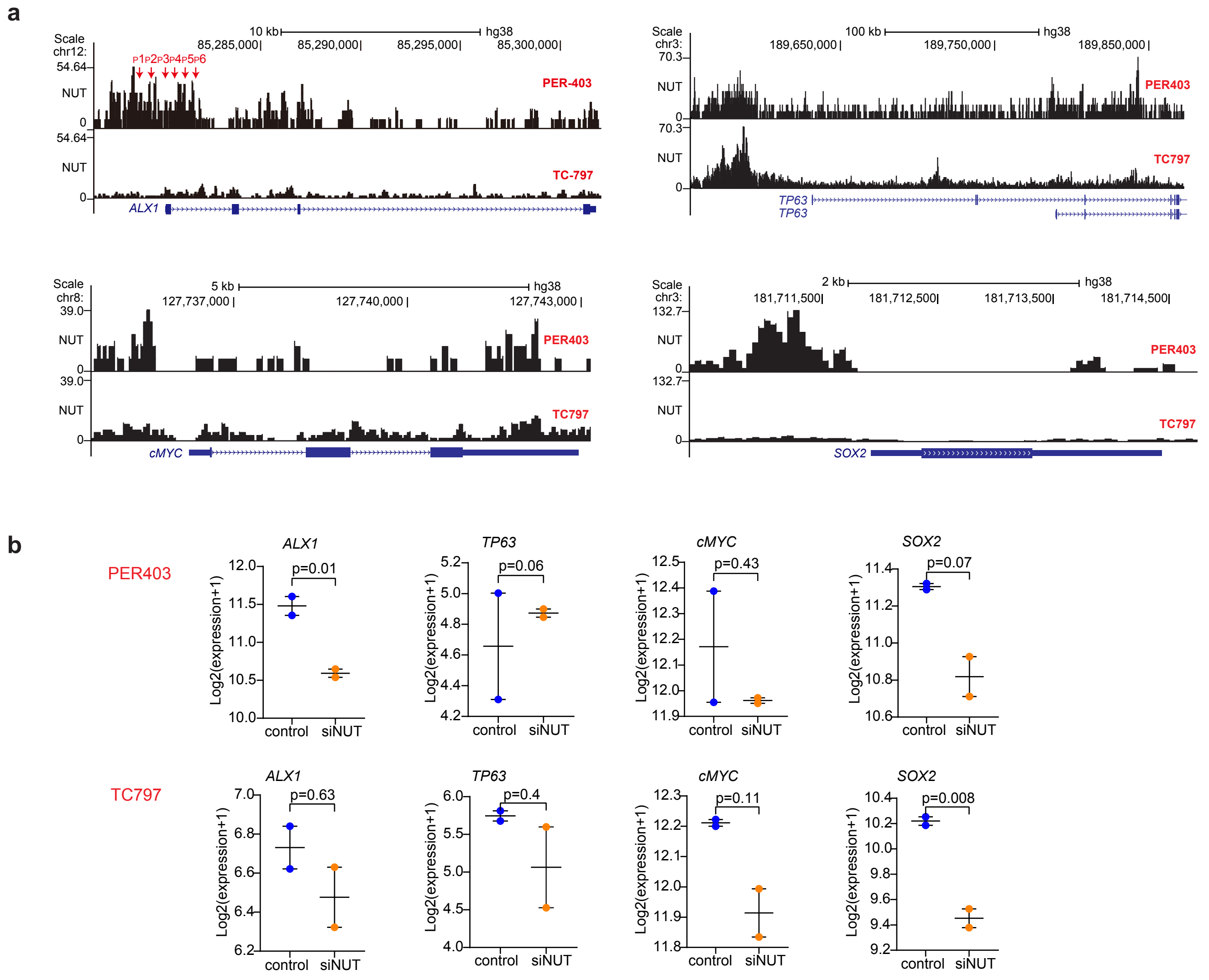

Supplementary Fig. 8 | Regulation of gene transcription by BRD4-NUT/p300 bipartite in NC cells. a, ChIP-seq tracks of BRD4-NUT at *ALX1*, *TP63*, *MYC* and *SOX2* loci in PER-403 (upper, GSE70870) and TC-797 cells (lower, GSE70870). b, RNA-seq analysis of *ALX1*, *TP63*, *MYC* and *SOX2* gene expressions in PER-403 (GSE18668) and TC-797 cells (GSE18668) transfected with indicated siRNA for NUT. Data represent mean ± SEM from two independent data sets. Two-sided p-values for each indicated comparison are derived from the R software loaded with Limma package.

Supplementary Table 1, NUT F1c Peptide Sequences

Peptides	Sequences
F1c (377-392)	WLVGTHLATGESDGKQ
F1c (403-418)	GMYPDPGLLSYINELC
F1c (403-418-mut)	GMYPDPGGGSGGNELC
F1c (419-434)	SQKVFVSKVEAVIHPQ
F1c (435-454)	FLADLLSPEKQRDPLALIEE
F1c (444-459)	KQRDPLALIEELEQEE
F1c (458-470)	EEGLTLAQLVQKR

Supplementary Table 2, Summary of the statistics of the final 20 NMR structures of the p300 TAZ2 domain in complex with NUT F1c peptide or TAZ2-NUT fusion protein.

	TAZ2/F1c	TAZ2-NUT
Protein NMR distance and dihedral constraints		
Distance constraints		
Total NOE	1879	2357
Intra-residue	853	1129
Inter-residue	1026	1228
Sequential (|i - j| = 1)	393	489
Medium-range (1 < |i - j| ≤ 5)	368	392
Long-range (|i - j| > 5)	265	347
Inter-molecular constraints	129	142
Hydrogen bonds	52	60
Total dihedral angle restraints		
Phi angle	83	97
Psi angle	83	97
Ramachandran Map Analysis (%)[a]		
Most favored regions	95.4	97.1
Additional allowed regions	4.6	2.9
Generally allowed regions	0.0	0.0
Disallowed regions	0.0	0.0
Structure statistics[c]		
Violations (mean +/- s.d.)		
Distance constraints (Å)	0.051 +/- 0.0059	0.057 +/- 0.0061
Dihedral angle constraints (°)	0.30 +/- 0.11	0.58 +/- 0.11
Max. dihedral angle violation (°)	0.60	0.91
Max. distance constraint violation (Å)	0.068	0.070
Deviations from idealized geometry		
Bond lengths (Å)	0.0048+/- 0.00015	0.0083+/- 0.00012
Bond angles (°)	0.64 +/- 0.022	0.66 +/- 0.018
Improper (°)	1.5 +/- 0.078	1.6 +/- 0.058
Average pairwise r.m.s. Deviation (Å)[b]		
Heavy	0.81 +/- 0.078	0.68 +/- 0.076
Backbone	0.40 +/- 0.095	0.33 +/- 0.084

[a] Procheck calculation was done for protein residues 8-34, 37-52, 59-78, 83-93 for TAZ2/F1c; 8-34, 37-52, 57-78, 83-93, 126-133, 140-153 for TAZ2-NUT.

[b] The residue number ranges used in full molecule pairwise root-mean-square (r.m.s.) deviation calculations consists of 10-93 for TAZ2/F1c; 10-92, 122-134, 140-150 for TAZ2-NUT.

[c] Pairwise r.m.s. deviation was calculated among top 20/200 lowest energy structures.

Supplementary Table 3, qPCR Primers

Primer Names	Primer sequence
SOX2-qPCR-F	GCTACAGCATGATGCAGGACCA
SOX2-qPCR-R	TCTGCGAGCTGGTCATGGAGTT
TP63-qPCR-F	CAGGAAGACAGAGTGTGCTGGT
TP63-qPCR-R	AATTGGACGGCGGTTCATCCCT
NBL1-qPCR-F	TCCACAGAGTCCCTGGTTCACT
NBL1-qPCR-R	GCTACAGTGCAGGATCTTCTCC
TGFBR2-qPCR-F	GTCTGTGGATGACCTGGCTAAC
TGFBR2-qPCR-R	GACATCGGTCTGCTTGAAGGAC
SRC-qPCR-F	CTGCTTTGGCGAGGTGTGGATG
SRC-qPCR-R	CCACAGCATACAACTGCACCAG
LOXL1-qPCR-F	ACAGCACCTGTGACTTCGGCAA
LOXL1-qPCR-R	CGGTTATGTCGATCCACTGGCA
ALX1-qPCR-F	TTGCCAAGGACTGACAGCTACC
ALX1-qPCR-R	GTCATACAGGAGGAAGTGTCACG
GAPDH-qPCR-F	GTCTCCTCTGACTTCAACAGCG
GAPDH-qPCR-R	ACCACCCTGTTGCTGTAGCCAA
SNAIL-qPCR-F	TGCCCTCAAGATGCACATCCGA
SNAIL-qPCR-R	GGGACAGGAGAAGGGCTTCT
N-cadherin-qPCR-F	CCTCCAGAGTTTACTGCCATGAC
N-cadherin-qPCR-R	GTAGGATCTCCGCCACTGATTC
E-cadherin-qPCR-F	GCCTCCTGAAAAGAGAGTGGAAG
E-cadherin-qPCR-R	TGGCAGTGTCTCTCCAAATCCG
Vimentin-qPCR-F	AGGCAAAGCAGGAGTCCACTGA
Vimentin-qPCR-R	ATCTGGCGTTCCAGGGACTCAT

Supplementary Table 4, *ALX1* ChIP-qPCR primers sequences

Primer Name	Primer Sequence
ALX1-ChIP-F1	AAATGGAGGCCTTGGACCTGTC
ALX1-ChIP-R1	GAGTTGCTGTGGAGTTACTCCGA
ALX1-ChIP-F2	AGGAGGCCAGCTGAGTTTCAAG
ALX1-ChIP-R2	AGGGAAGGGAAAGCGAGCG
ALX1-ChIP-F3	GAGCCCTCCGAGTAAAAACAGTGAC
ALX1-ChIP-R3	TGCAGACGCTTTGCTGTAAAAGGAC
ALX1-ChIP-F4	GTACGTCACTGTCGTCACGAGT
ALX1-ChIP-R4	CGCCACGTCTTGGAAAATTAAAAAGA
ALX1-ChIP-F5	GCGTTTGCAATACAACACGCGT
ALX1-ChIP-R5	GAGTGATTAGGCCGGGGATGGT
ALX1-ChIP-F6	GCGACAGCTAAAGCACGTTTACAATT
ALX1-ChIP-R6	CAGGGGGCTTGTTGTAAATATTTCACAC
