# Supplementary material for: Structural mechanism of BRD4-NUT and p300 bipartite interaction in propagating aberrant gene transcription in chromatin in NUT carcinoma
Source: Nat Commun. 2023 Jan 24;14:378. doi: 10.1038/s41467-023-36063-5 (PMC9870903; doi:10.1038/s41467-023-36063-5)
Supplement: Supplementary file 3 — Reporting Summary [file 41467_2023_36063_MOESM3_ESM.pdf]

nature portfolio

Corresponding author(s): Ming-MIng Zhou, Lei Zeng

Last updated by author(s): Dec 26, 2022

Reporting Summary

Nature Portfolio wishes to improve the reproducibility of the work that we publish. This form provides structure for consistency and transparency in reporting. For further information on Nature Portfolio policies, see our Editorial Policies and the Editorial Policy Checklist.

Statistics

For all statistical analyses, confirm that the following items are present in the figure legend, table legend, main text, or Methods section.

n/a	Confirmed	
☐	☒	The exact sample size (n) for each experimental group/condition, given as a discrete number and unit of measurement
☐	☒	A statement on whether measurements were taken from distinct samples or whether the same sample was measured repeatedly
☐	☒	The statistical test(s) used AND whether they are one- or two-sided<br>*Only common tests should be described solely by name; describe more complex techniques in the Methods section.*
☒	☐	A description of all covariates tested
☒	☐	A description of any assumptions or corrections, such as tests of normality and adjustment for multiple comparisons
☐	☒	A full description of the statistical parameters including central tendency (e.g. means) or other basic estimates (e.g. regression coefficient) AND variation (e.g. standard deviation) or associated estimates of uncertainty (e.g. confidence intervals)
☐	☒	For null hypothesis testing, the test statistic (e.g. F, t, r) with confidence intervals, effect sizes, degrees of freedom and P value noted<br>*Give P values as exact values whenever suitable.*
☒	☐	For Bayesian analysis, information on the choice of priors and Markov chain Monte Carlo settings
☒	☐	For hierarchical and complex designs, identification of the appropriate level for tests and full reporting of outcomes
☐	☒	Estimates of effect sizes (e.g. Cohen's d, Pearson's r), indicating how they were calculated

Our web collection on statistics for biologists contains articles on many of the points above.

Software and code

Policy information about availability of computer code

Data collection	ZEN 2012 (Blue Edition), OLYMPUS cellSens Standard v1.18, LightCycler 480 Software v1.5.1.62, topspin v3.0
Data analysis	ImageJ (1.48V), GraphPad Prism (v6.0c) and Excel for Mac (v14.3.0), NMRPipe v2.0, NMRVIEW v5.0, CNS v1.5, ARIA v2.0, PROCHECK v.3.5.4, TALOS+ v3.70F1, PONDR VSL2 (http://www.pondr.com/), PSRSM (http://qilubio.qlu.edu.cn:82/protein_PSRSM/default.aspx), R software loaded with Limma package (R software v3.2.3, Limma package v3.26.8).

For manuscripts utilizing custom algorithms or software that are central to the research but not yet described in published literature, software must be made available to editors and reviewers. We strongly encourage code deposition in a community repository (e.g. GitHub). See the Nature Portfolio guidelines for submitting code & software for further information.

Data

Policy information about availability of data

All manuscripts must include a data availability statement. This statement should provide the following information, where applicable:

- Accession codes, unique identifiers, or web links for publicly available datasets
- A description of any restrictions on data availability
- For clinical datasets or third party data, please ensure that the statement adheres to our policy

Data availability

The data that support this study are available from the corresponding author upon reasonable request. The Structure coordinate data for the p300 TAZ2 domain in

complex with NUT TADs peptides generated in this study have been deposited in the Protein Data Bank database under PDB accession code 7XEZ and 7XFG [https://www.rcsb.org]. The NMR data are being deposited in the Biological Magnetic Resonance Data Bank (BMRB code 36480 and 36481) [https://bmrb.io]. All protocols used in this study are available in the Methods sections. The source data for figures in the study are provided in separate Excel and PDF files, and they are protected and are not available due to data privacy laws. Supplementary Information is available in the online version of the paper.

Human research participants

Policy information about studies involving human research participants and Sex and Gender in Research.

Reporting on sex and gender	NA
Population characteristics	NA
Recruitment	NA
Ethics oversight	NA

Note that full information on the approval of the study protocol must also be provided in the manuscript.

Field-specific reporting

Please select the one below that is the best fit for your research. If you are not sure, read the appropriate sections before making your selection.

☒ Life sciences ☐ Behavioural & social sciences ☐ Ecological, evolutionary & environmental sciences

For a reference copy of the document with all sections, see nature.com/documents/nr-reporting-summary-flat.pdf

Life sciences study design

All studies must disclose on these points even when the disclosure is negative.

Sample size	The sample size of each experiment and statistical analysis have been shown in the legend, and these sample sizes was ensured to detect differences between experimental groups.
Data exclusions	No data exclusions
Replication	All in vitro and cellular experiments were independently replicated at least twice and repeated at least three times within each of the experimental runs.
Randomization	In cellular and in-vitro confocal photography, liquid droplets and nuclear puncta were randomized. Cellular experiments were involved in random transfection grouping.
Blinding	Blinding was not performed in this study, this is because this study was not applied in animal studies, immunoassays and clinical trials.

Reporting for specific materials, systems and methods

We require information from authors about some types of materials, experimental systems and methods used in many studies. Here, indicate whether each material, system or method listed is relevant to your study. If you are not sure if a list item applies to your research, read the appropriate section before selecting a response.

Materials & experimental systems

n/a	Involved in the study	
☐	☒	Antibodies
☐	☒	Eukaryotic cell lines
☒	☐	Palaeontology and archaeology
☒	☐	Animals and other organisms
☒	☐	Clinical data
☒	☐	Dual use research of concern

Methods

n/a	Involved in the study	
☒	☐	ChIP-seq
☒	☐	Flow cytometry
☒	☐	MRI-based neuroimaging

Antibodies

Antibodies used	1. Rabbit Monoclonal anti-GAPDH, Abcam, Cat# ab181602, 1:5000 WB dilution;

2. Rabbit Monoclonal anti-Histone H3 (acetyl K18), Abcam, Cat# ab40888, 1:10000 WB dilution, 1:1000 IF dilution;
3. Rabbit Monoclonal anti-Histone H3 (acetyl K27), Abcam, Cat# ab4729, 1:2000 WB dilution, 1:2000 IF dilution, 1:500 ChIP dilution;
4. Rabbit Monoclonal anti-Histone H3 (acetyl K56), Abcam, Cat# ab71956, 1:3000 WB dilution;
5. Rabbit Monoclonal anti-Histone H3, Abcam, Cat# ab1791, 1:2000 WB dilution;
6. Rabbit Monoclonal anti-KAT3B/p300, Abcam, Cat# ab54984, 1:2000 WB dilution;
7. Mouse Monoclonal anti-p53, Abcam, Cat# ab1101, 1:2000 WB dilution;
8. Rabbit Polyclonal anti-TRAP220/MED1, Abcam, Cat# ab64965, 1:500 IF dilution;
9. Rabbit Polyclonal anti-CDK9, Abcam, Cat# ab6544, 1:600 IF dilution;
10. Goat Polyclonal anti-SNAIL, Abcam, Cat# ab53519, 1:2000 WB dilution;
11. Rabbit Monoclonal anti-Alx1, Abcam, Cat# ab181101, 1:2000 WB dilution;
12. Rabbit Polyclonal anti-RNA polymerase II CTD repeats YSPTSPS (phospho S5), Abcam, Cat# ab5131, 1:1000 IF Dilution;
13. Rabbit Polyclonal anti-RNA polymerase II CTD repeats YSPTSPS (phospho S2), Abcam, Cat# ab5095, 1:500 IF dilution;
14. Acetyl-Histone H4 (Lys12) (D2W6O) Rabbit mAb, CST, Cat# 13944, 1:1000 WB dilution;
15. Acetylated-Lysine Antibody, Rabbit, CST, Cat# 9441, 1:1000 WB dilution;
16. E-Cadherin (4A2) Mouse mAb, CST, Cat# 14472, 1:1000 WB dilution;
17. Vimentin (D21H3) XP® Rabbit mAb, CST, CAT# 5741, 1:1000 WB dilution;
18. N-Cadherin (D4R1H) XP® Rabbit mAb, CST, Cat# 13116, 1:1000 WB Dilution;
19. Rabbit Polyclonal Anti-acetyl-p53 Antibody (Lys373, Lys382), Merck Millipore, Cat# 06-758, 1:2000 WB dilution;
20. Monoclonal Anti-Involucrin antibody produced in mouse, Sigma-Aldrich, Cat# i9018, 1:1000 WB dilution;
21. Anti-GFP tag Mouse mAb, Engibody, Cat# AT0028, 1:2000 WB dilution;
22. GFP-tag Antibody Mouse mAb, Affinity, Cat# T0005, 1:2000 WB dilution;
23. Mouse Monoclonal anti-MBP tag, Proteintech, Cat# 66003, 1:2000 WB dilution;
24. Mouse monoclonal ANTI-FLAG® M2 antibody, Merck Millipore, Cat# F1804, 1:1000 IF dilution, 1:100 ChIP dilution;
25. Goat anti-Mouse IgG (H+L) Cross-Adsorbed Secondary Antibody, Alexa Fluor™ 594, Invitrogen, Cat# A-11005, 1:1000 IF dilution;
26. Goat anti-Rabbit IgG (H+L) Cross-Adsorbed Secondary Antibody, Alexa Fluor™ 594, Invitrogen, Cat# A-11012, 1:1000 IF dilution;
27. Rabbit Polyclonal anti-BRD4, Bethyl, Cat# A301-985A100, 1:100 ChIP dilution;
28. Mouse Monoclonal anti-KAT3B/p300, Abcam, Cat# ab14984, 1:100 ChIP dilution, 1:500 IF dilution;
29. Normal mouse IgG, Santa Cruz, Cat# sc-2025, 1:200 ChIP dilution;
30. Normal rabbit IgG, CST, Cat #2729, 1:200 ChIP dilution;
31. Goat Anti-Rabbit IgG H&L (HRP), Abcam, Cat# ab6721, 1:10000 WB dilution;
32. Goat Anti-Mouse IgG H&L (HRP), Abcam, Cat# ab205719, 1:10000 WB dilution;
33. Rabbit Anti-Goat IgG H&L (HRP), Abcam, Cat# ab6741, 1:5000 WB dilution.

Validation

1. https://www.abcam.com/gapdh-antibody-epr16891-loading-control-ab181602.html;
2. https://www.abcam.com/histone-h3-acetyl-k18-antibody-ep959y-chip-grade-ab40888.html;
3. https://www.abcam.com/histone-h3-acetyl-k27-antibody-chip-grade-ab4729.html;
4. https://www.abcam.com/ab71956.pdf?;
5. https://www.abcam.com/histone-h3-antibody-nuclear-marker-and-chip-grade-ab1791.html;
6. https://www.abcam.com/ab54984.pdf?;
7. https://www.abcam.com/p53-antibody-do-1-chip-grade-ab1101.html;
8. https://www.abcam.com/trap220med1-antibody-ab64965.html;
9. https://www.abcam.com/cdk9-antibody-ab6544.html;
10. https://www.abcam.com/ab53519.pdf?;
11. https://www.abcam.com/alx1-antibody-epr11331-ab181101.html;
12. https://www.abcam.com/rna-polymerase-ii-ctd-repeat-ysptsps-phospho-s5-antibody-ab5131.html;
13. https://www.abcam.com/rna-polymerase-ii-ctd-repeat-ysptsps-phospho-s2-antibody-ab5095.html;
14. https://www.cellsignal.com/products/primary-antibodies/acetyl-histone-h4-lys12-d2w6o-rabbit-mab/13944;
15. https://www.cellsignal.com/products/primary-antibodies/acetylated-lysine-antibody/9441;
16. https://www.cellsignal.com/products/primary-antibodies/e-cadherin-4a2-mouse-mab/14472;
17. https://www.cellsignal.com/products/primary-antibodies/vimentin-d21h3-xp-rabbit-mab/5741?_=1672099610266&Ntt=5741&tahead=true;
18. https://www.cellsignal.com/products/primary-antibodies/n-cadherin-d4r1h-xp-rabbit-mab/13116?_=1672099699162&Ntt=13116&tahead=true;
19. https://www.emdmillipore.com/US/en/product/Anti-acetyl-p53-Antibody-Lys373-Lys382,MM_NF-06-758?bd=1;
20. https://www.sigmaaldrich.cn/CN/en/product/sigma/i9018
21. http://www.engibody.com/products/GFP-tag-mouse-mab-Epitope-Tag-Antibody-at0028.html;
22. https://affbiotech.com/goods-6271-T0005-GFP_tag_Antibody.html;
23. https://www.ptglab.com/Products/MBP-Tag-Antibody-66003-1-Ig.htm;
24. https://www.sigmaaldrich.cn/CN/en/product/sigma/f1804;
25. https://www.thermofisher.cn/cn/en/antibody/product/Goat-anti-Mouse-IgG-H-L-Cross-Adsorbed-Secondary-Antibody-Polyclonal/A-11005;
26. https://www.thermofisher.cn/cn/zh/antibody/product/Goat-anti-Rabbit-IgG-H-L-Cross-Adsorbed-Secondary-Antibody-Polyclonal/A-11012;
27. https://www.thermofisher.cn/cn/en/antibody/product/BRD4-Antibody-Polyclonal/A301-985A100;
28. https://www.abcam.com/kat3b--p300-antibody-3g230--nm-11-chip-grade-ab14984.html;
29. https://www.scbt.com/p/normal-mouse-igg?requestFrom=search;
30. https://www.cellsignal.com/products/primary-antibodies/normal-rabbit-igg/2729;
31. https://www.abcam.com/goat-rabbit-igg-hl-hrp-ab6721.html;
32. https://www.abcam.com/goat-mouse-igg-hl-hrp-ab205719.html;

33. https://www.abcam.com/rabbit-goat-igg-hl-hrp-ab6741.html.

Eukaryotic cell lines

Policy information about cell lines and Sex and Gender in Research

Cell line source(s)	HEK293 and LO2 cell lines were from ATCC, NC 14169 cell line was from Christopher A French at Harvard Medical School
Authentication	The cell lines were authenticated via short tandem repeat (STR) profiling by the American Type Culture Collection (ATCC) and C.A. French.
Mycoplasma contamination	None of the cell line used had been tested for mycoplasma contamination.
Commonly misidentified lines (See ICLAC register)	No commonly misidentified cell lines were used in this study.
