# Supplementary material for: Structural mechanism of BRD4-NUT and p300 bipartite interaction in propagating aberrant gene transcription in chromatin in NUT carcinoma
Source: Nat Commun. 2023 Jan 24;14:378. doi: 10.1038/s41467-023-36063-5 (PMC9870903; doi:10.1038/s41467-023-36063-5)

Fig 1D

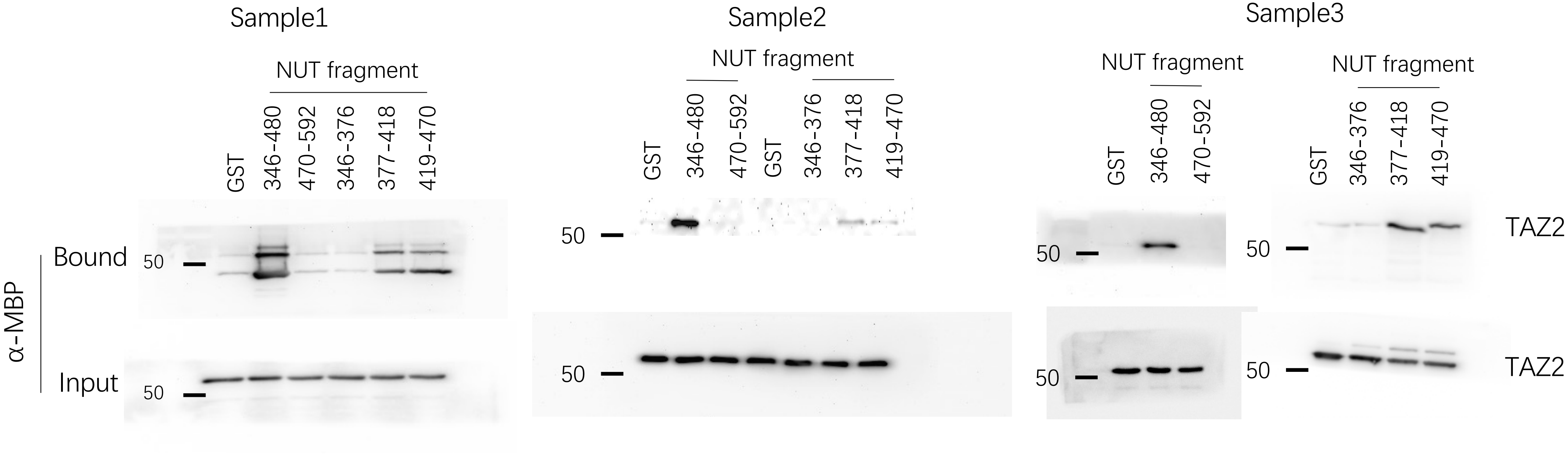

Bound/Input	GST	346-480	470-592	346-376	377-418	419-470
1	1.00	21.81	0.99	1.06	10.82	10.13
2	1.00	18.88	0.86	1.06	11.82	10.82
3	1.00	29.49	1.25	0.73	30.53	19.30
Ave	1.00	23.39	1.03	0.95	17.72	13.42
P value	NA	0.0021	NA	NA	0.0595	0.0136

Fig 1D

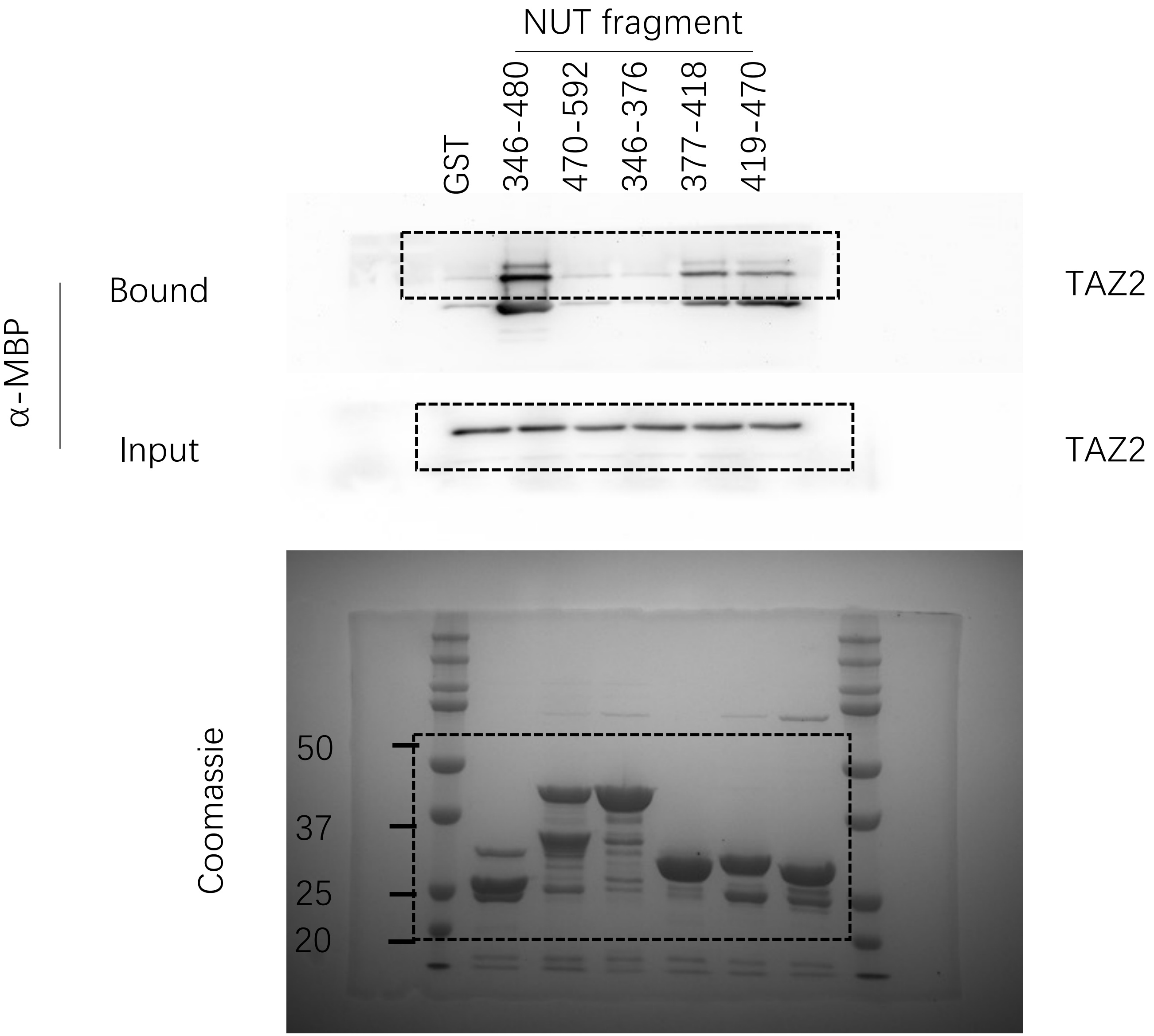
NUT fragment
GST
346-480
470-592
346-376
377-418
419-470
α-MBP
Bound
Input
TAZ2
TAZ2
Coomassie
50
37
25
20

Fig 1G

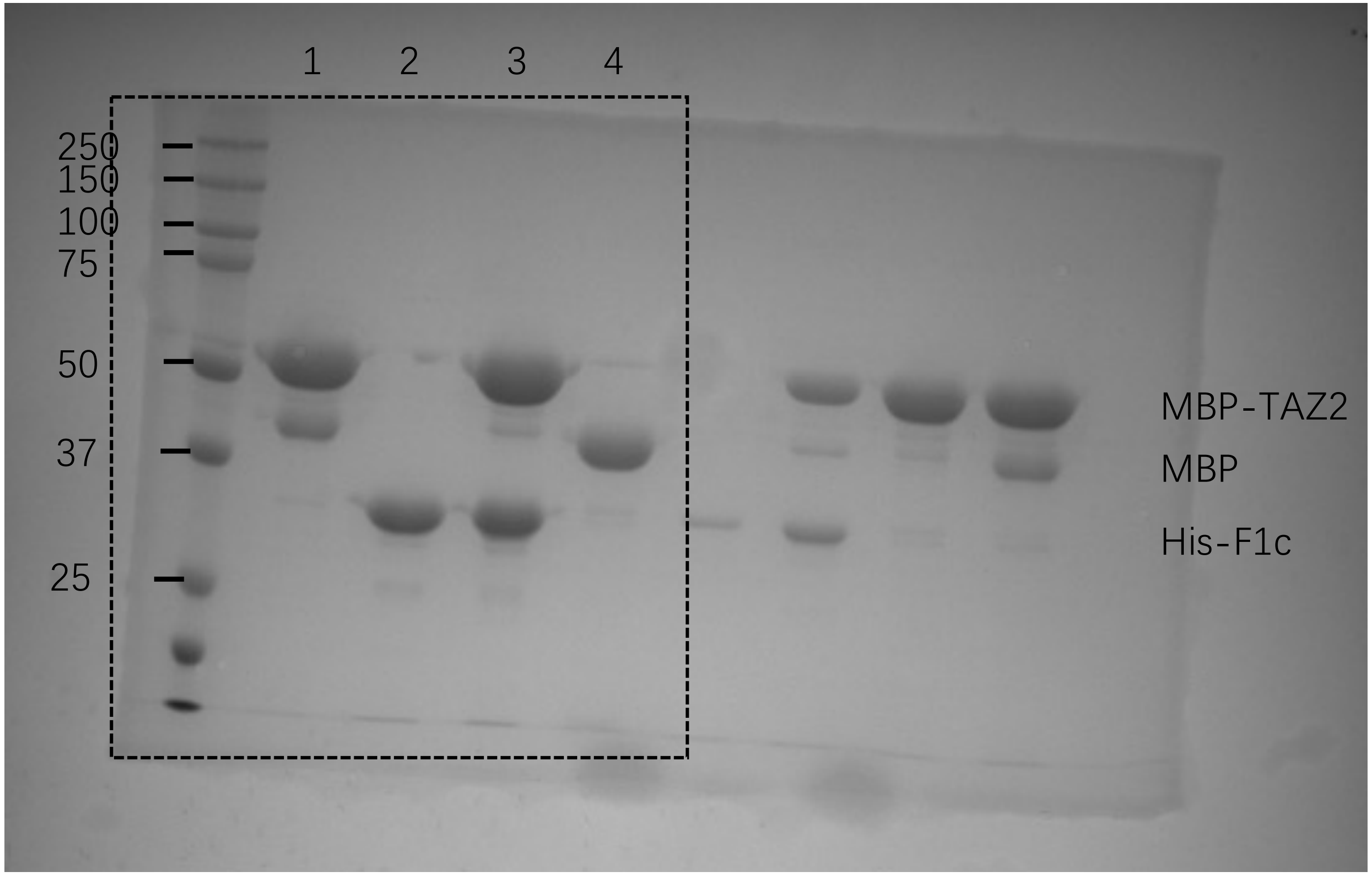

Fig 3E

sample1

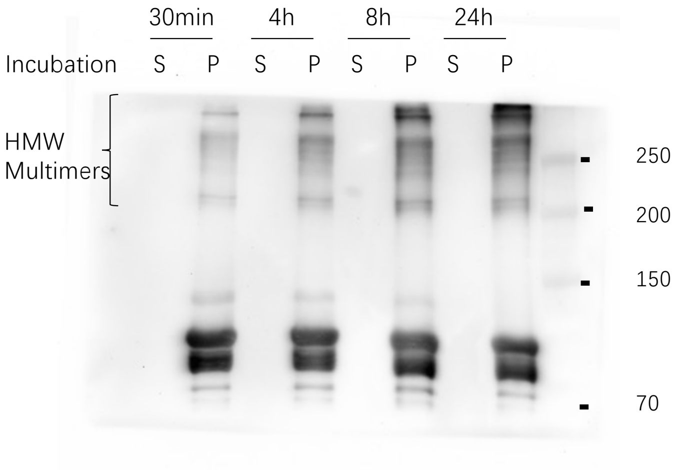

sample2

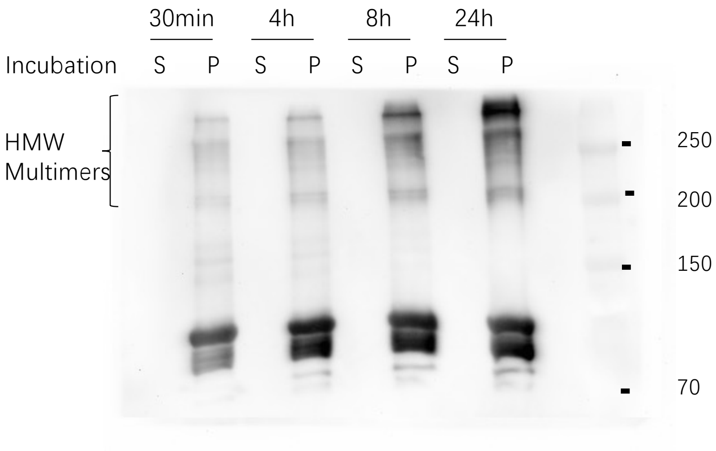

sample3

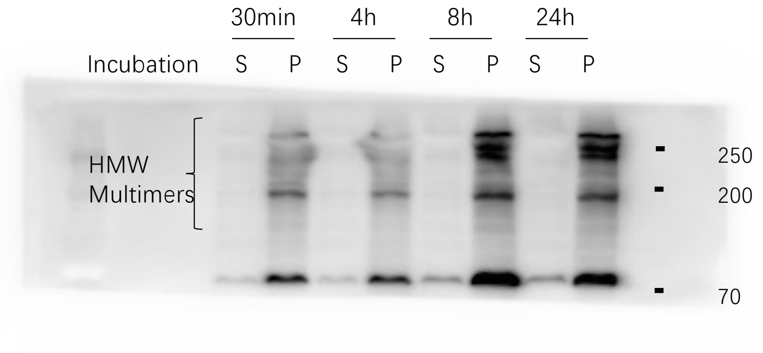

Intensity	30min	4h	8h	24h
1	10215.64	21420.04	33874.5	41105.27
2	22951.06	26215.34	43782.49	57609.92
3	16213.35	23934.69	38619.49	49660.6
ave	16460.0167	23856.69	38758.8267	49458.5967
P value	NA	0.1393	0.0088	0.0055

P300

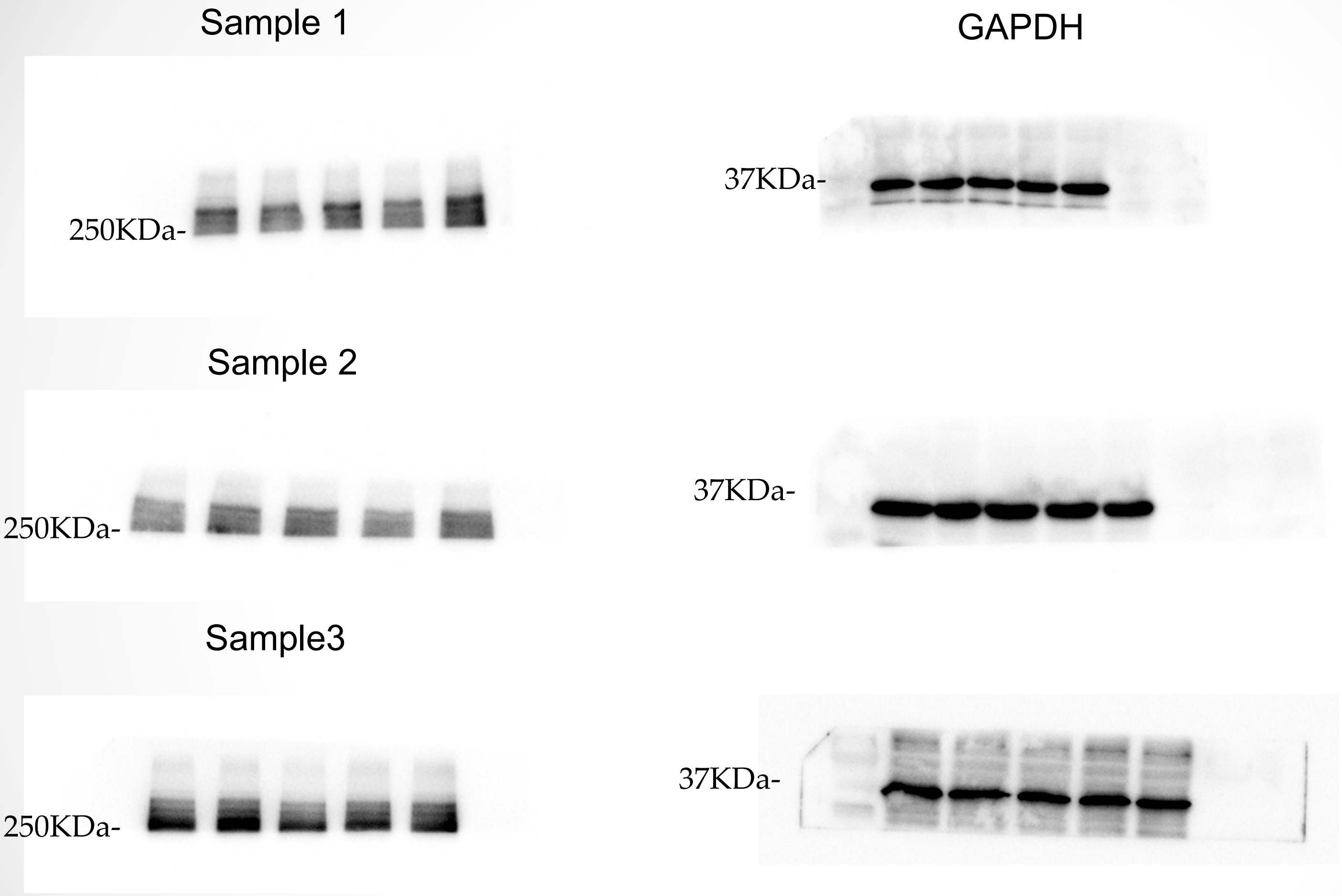

P300	EV	WT	ΔTAD1	ΔTAD2	ΔF1C
1	1.00	0.83	0.81	0.97	0.92
2	1.00	1.34	1.27	1.15	1.35
3	1.00	1.14	0.88	1.12	1.15
P value		0.5187	0.6005	0.8930	0.8586
Ave	1	1.10	0.99	1.08	1.14

Figure 5a

P300ac

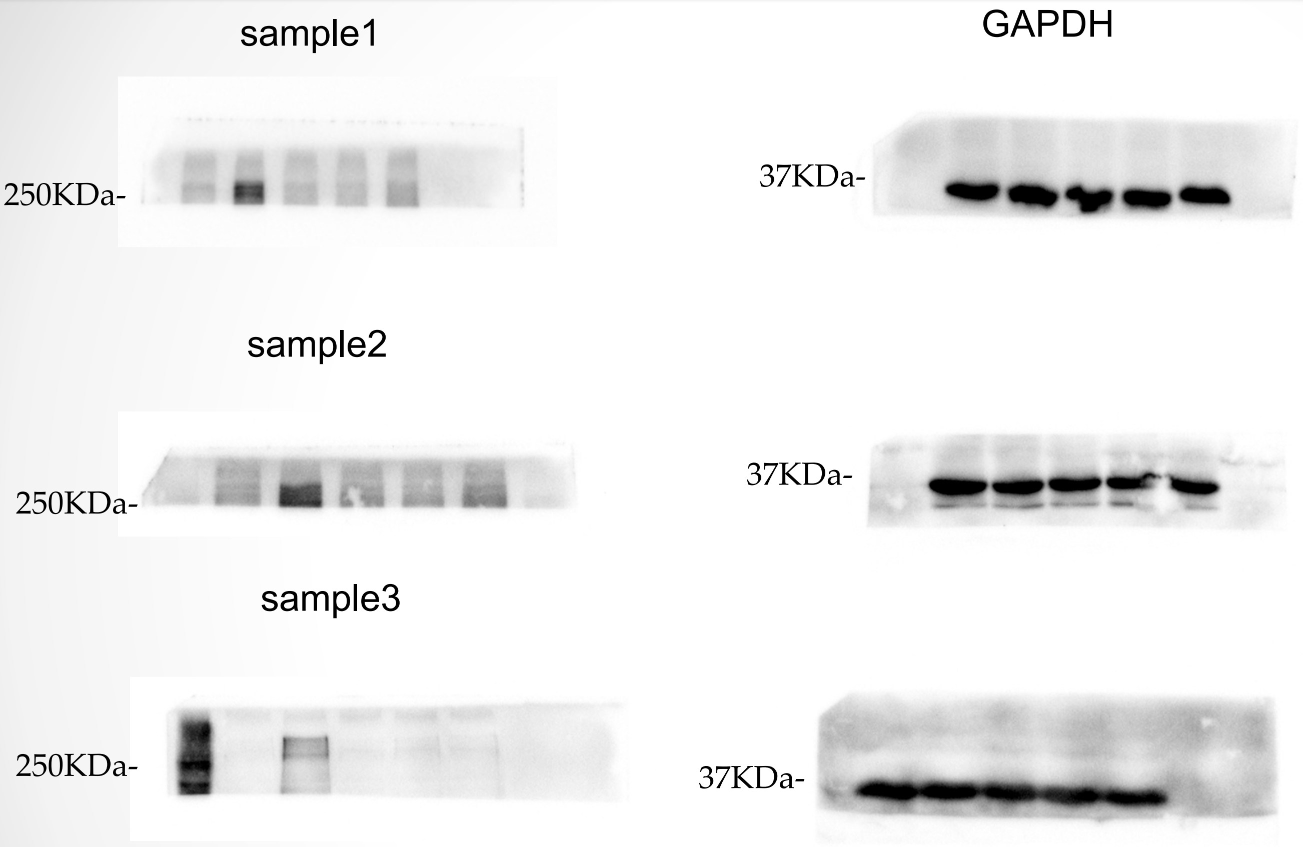

P300AC	EV	WT	ΔTAD1	ΔTAD2	ΔF1C
1	1.00	2.66	0.92	1.34	0.92
2	1.00	2.61	0.99	1.18	0.94
3	1.00	1.97	1.09	1.20	1.19
P value		0.0032	0.0035	0.0068	0.0043
Ave	1	2.41	1.00	1.24	1.02

Figure 5a

H3K27ac

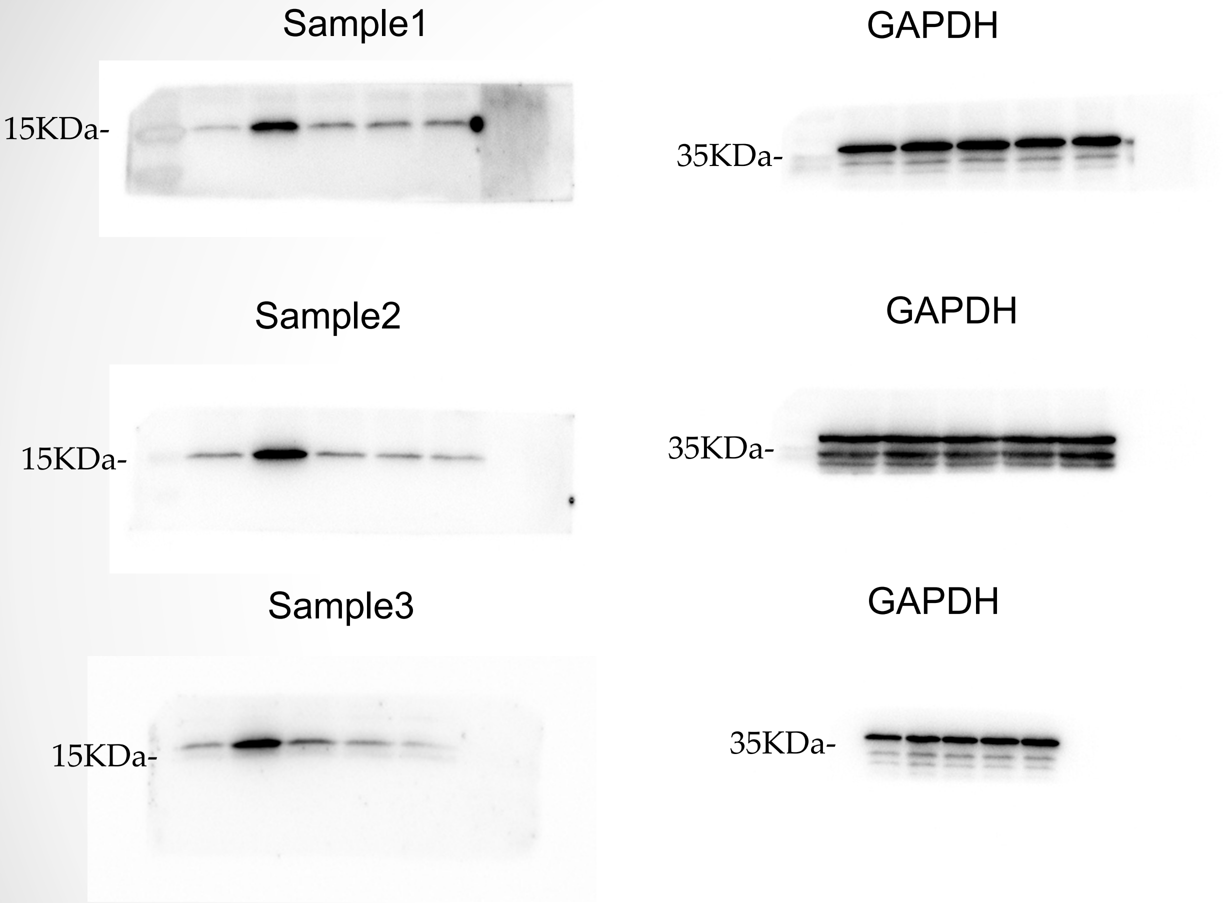

H3K27AC	EV	WT	ΔTAD1	ΔTAD2	ΔF1C
1	1.00	5.47	1.96	2.09	2.09
2	1.00	4.70	1.42	1.15	1.09
3	1.00	5.52	2.52	1.26	0.81
P value		0.0001	0.0014	0.0007	0.0012
Ave	1	5.23	1.97	1.50	1.33

Figure 5a

H3K56ac

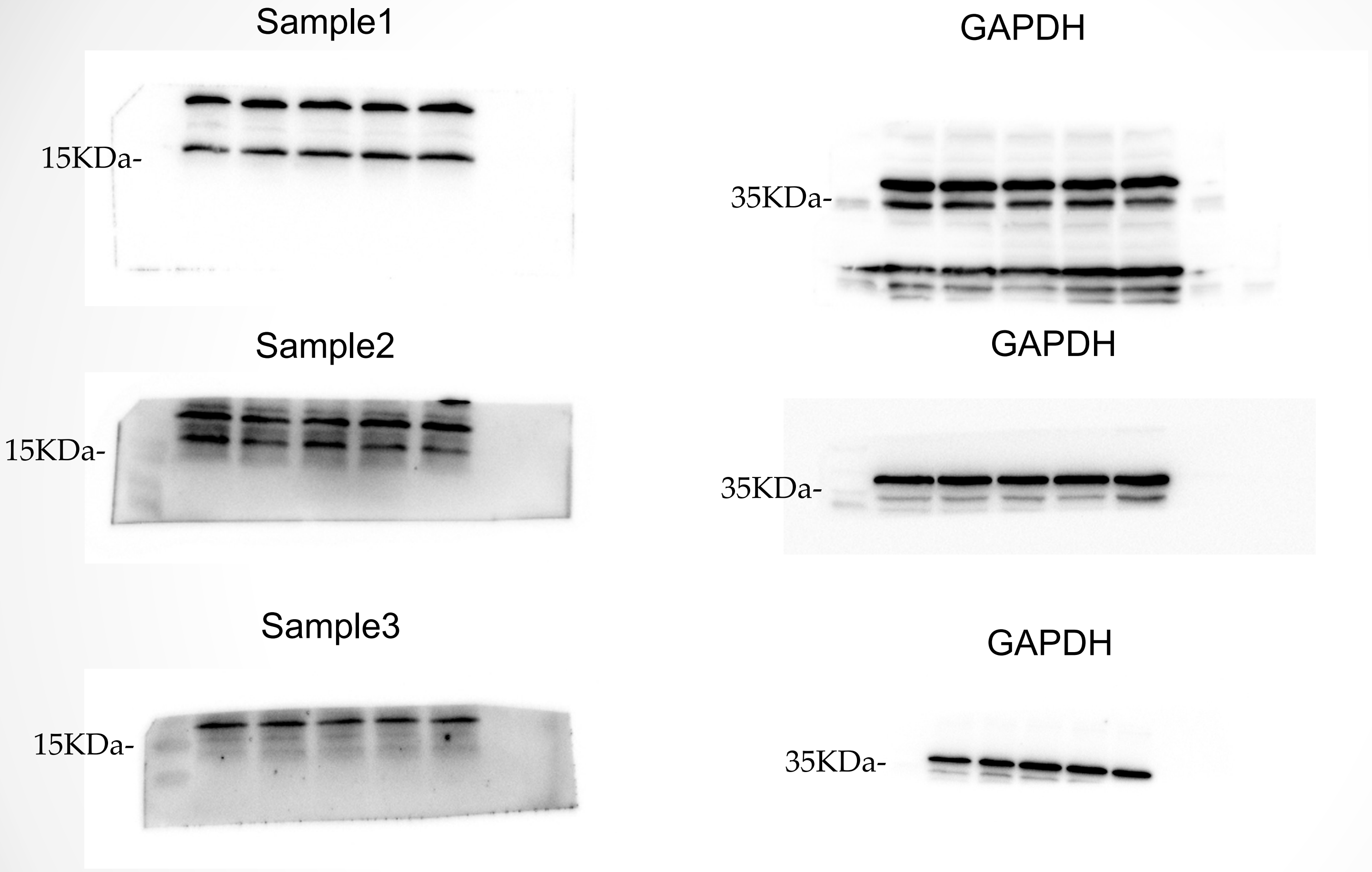

H3K56AC	EV	WT	ΔTAD1	ΔTAD2	ΔF1C
1	1.00	1.09	1.30	1.40	1.40
2	1.00	0.94	0.96	0.87	0.85
3	1.00	1.04	0.92	0.85	0.93
P value		0.5885	0.7981	0.9573	0.8643
Ave	1	1.03	1.06	1.04	1.06

Figure 5a

H3K18ac

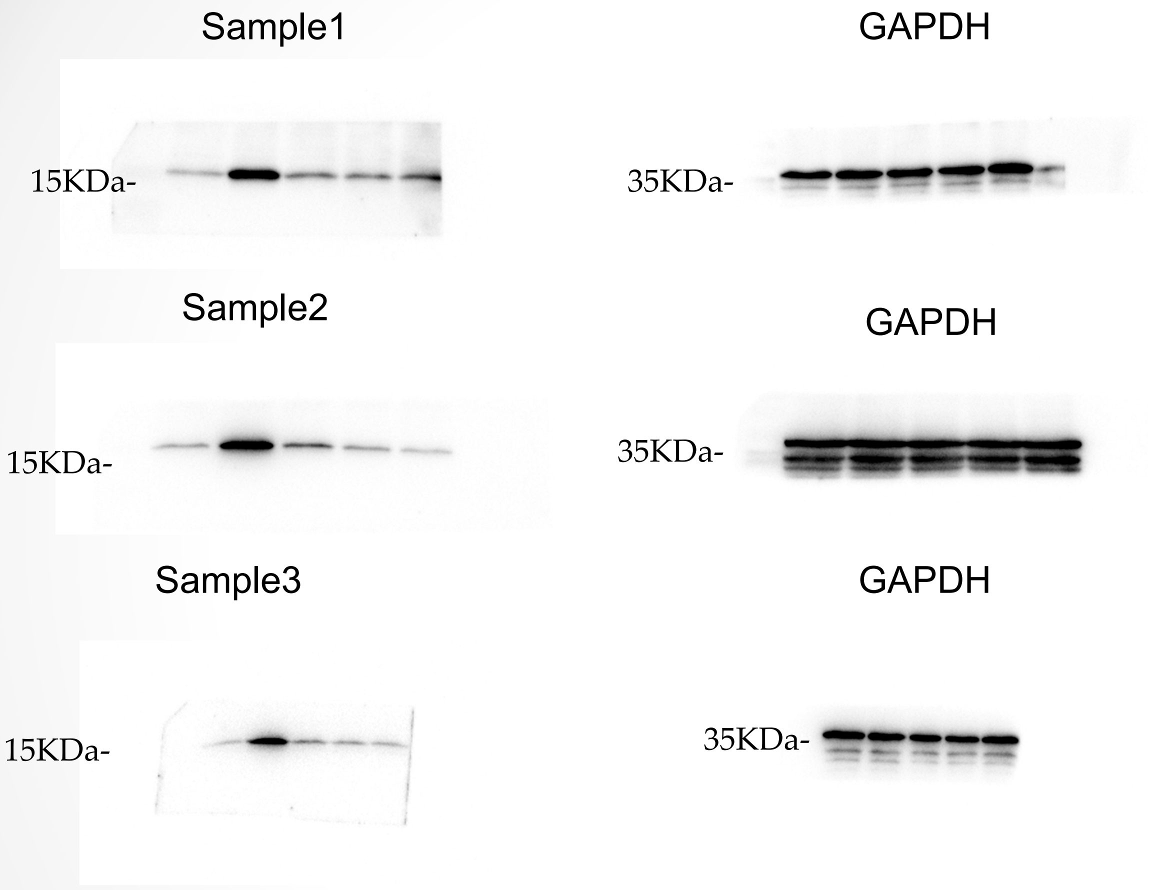

H3K18AC	EV	WT	ΔTAD1	ΔTAD2	ΔF1C
1	1.00	5.96	2.03	1.09	0.79
2	1.00	4.88	1.68	1.47	2.13
3	1.00	5.44	2.30	1.88	1.34
P value		0.0001	0.0007	0.0005	0.0013
Ave	1	5.43	2.00	1.48	1.42

Figure 5a

H4K12ac

Sample1

15KDa-

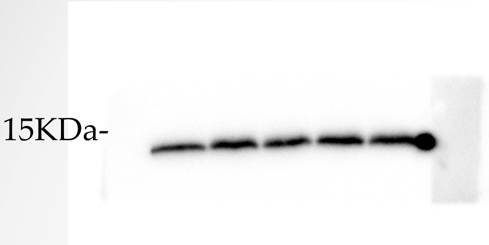

GAPDH

35KDa-

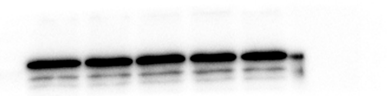

Sample2

15KDa-

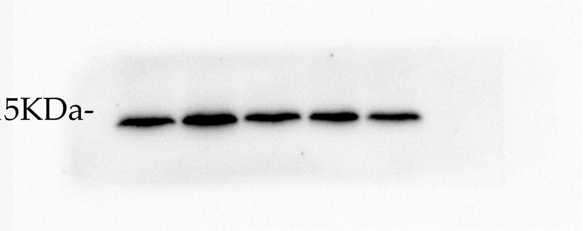

GAPDH

35KDa-

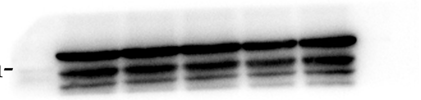

Sample 3

15KDa-

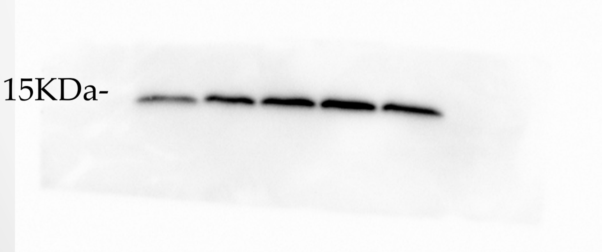

GAPDH

35KDa-

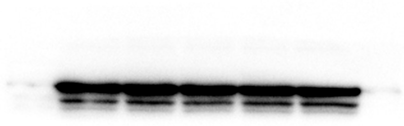

H4K12AC	EV	WT	ΔTAD1	ΔTAD2	ΔF1C
1	1.00	1.07	1.03	1.11	1.06
2	1.00	1.25	1.07	1.03	0.74
3	1.00	0.76	0.72	0.77	0.76
P value		0.8500	0.6538	0.7505	0.3780
Ave	1	1.03	0.94	0.97	0.85

Figure 5a

BRD4-NUT

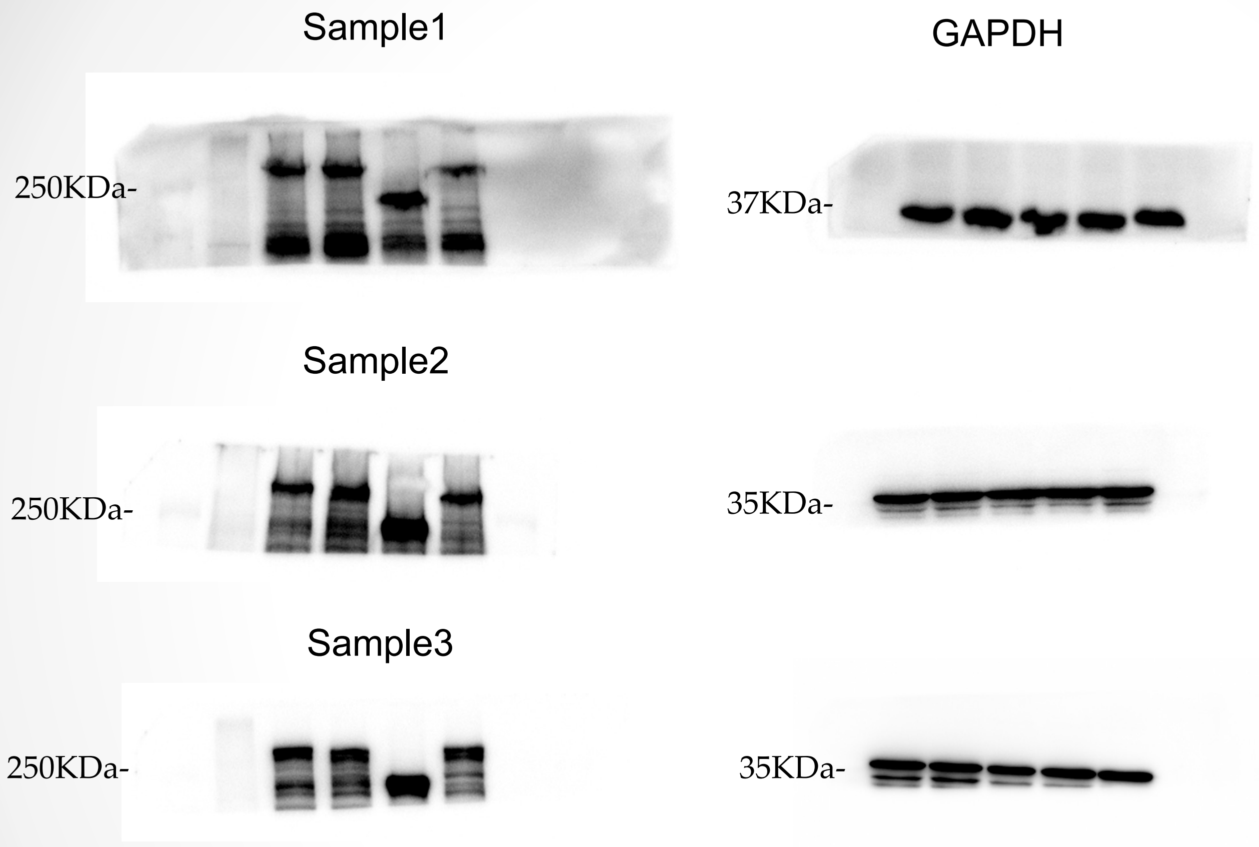

Figure 5b

p300

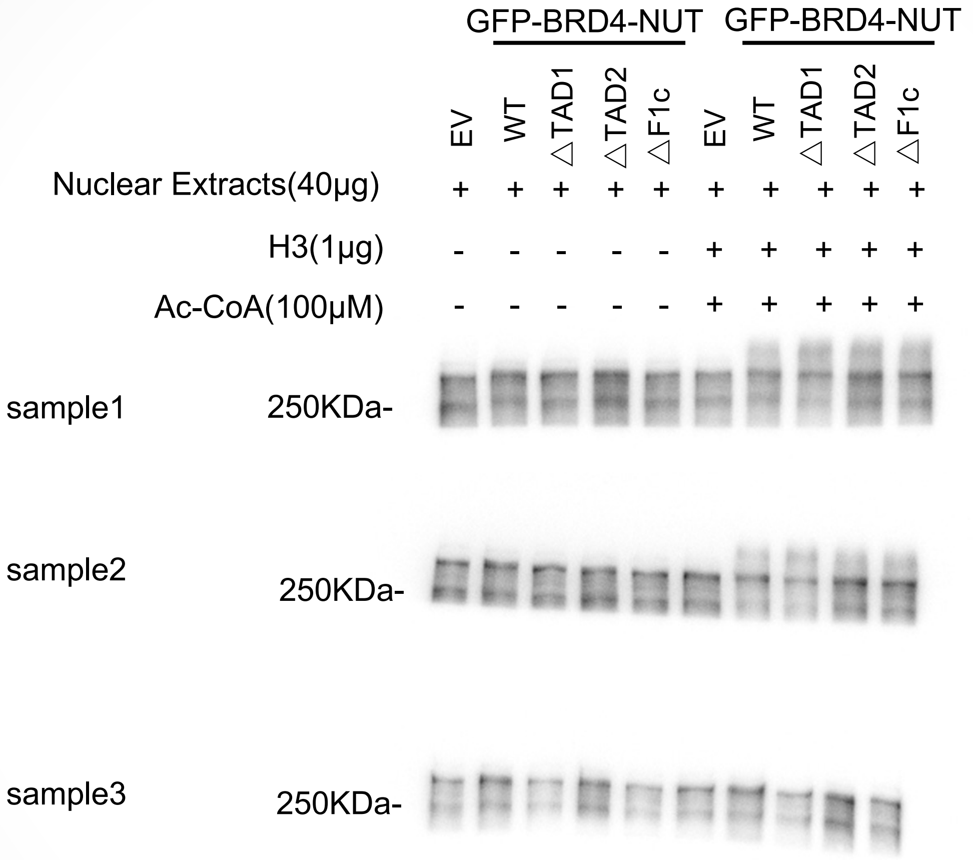

Figure 5b

p300ac

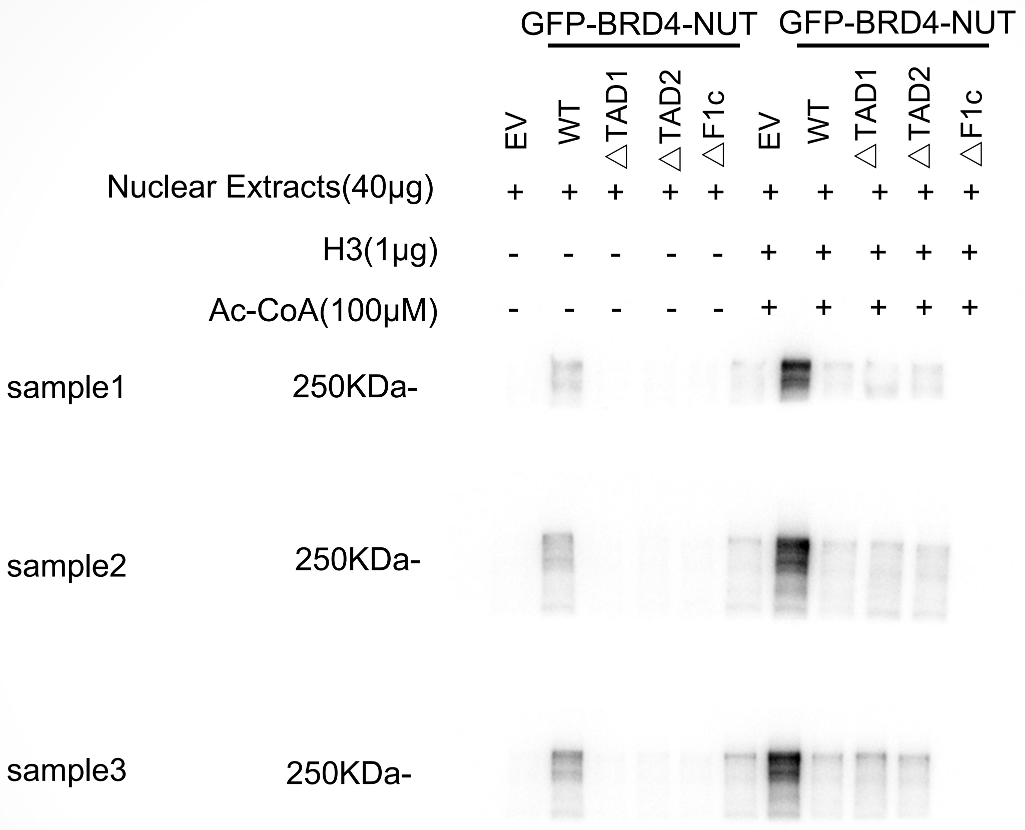

p300ac	1.00	12.91	1.72	0.90	1.19
	1.00	14.74	1.19	1.24	1.15
	1.00	14.22	1.93	2.28	2.23
Ave	1.00	13.95	1.61	1.47	1.52
P value		0.0000	0.0000	0.0001	0.0000

Figure 5b

BRD4-NUT

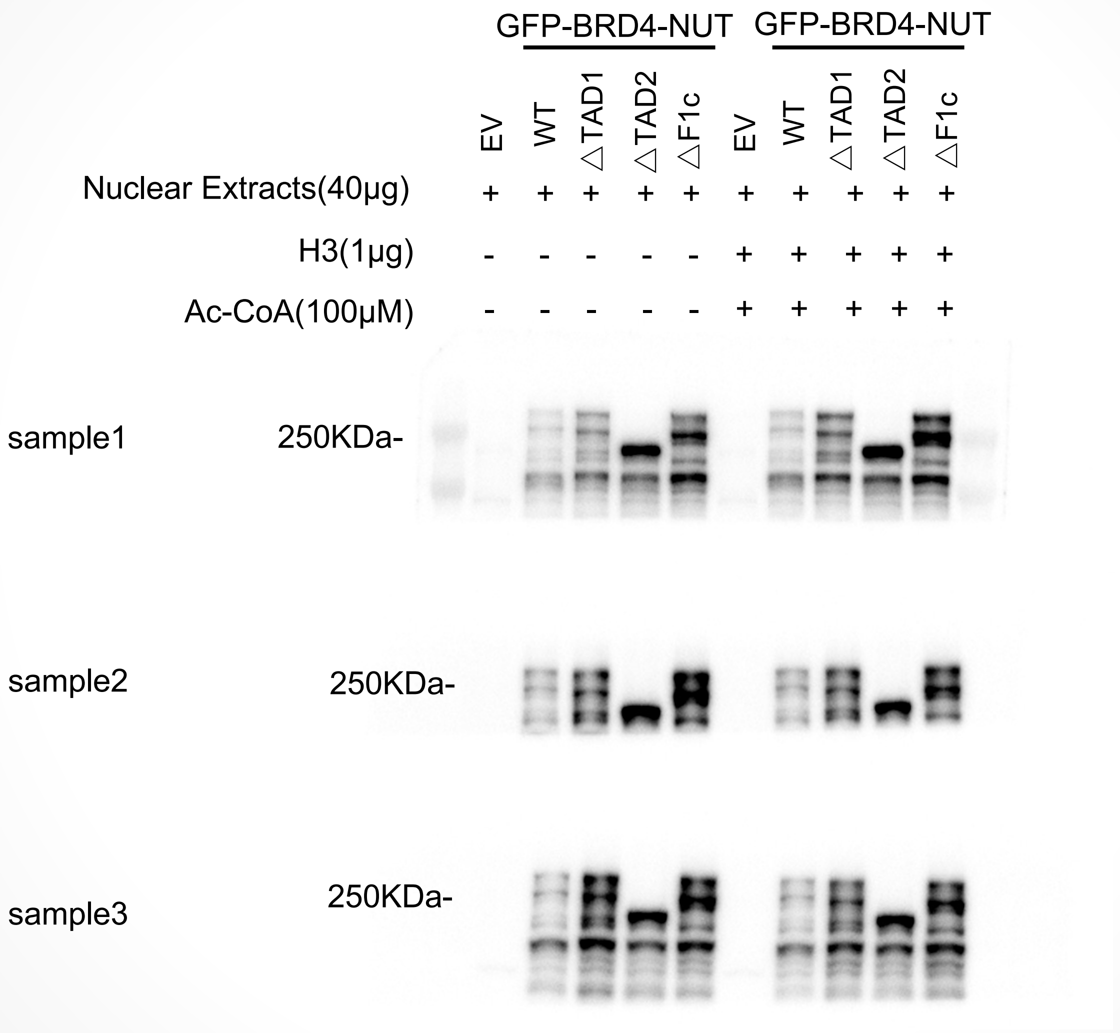

Figure 5b

H3

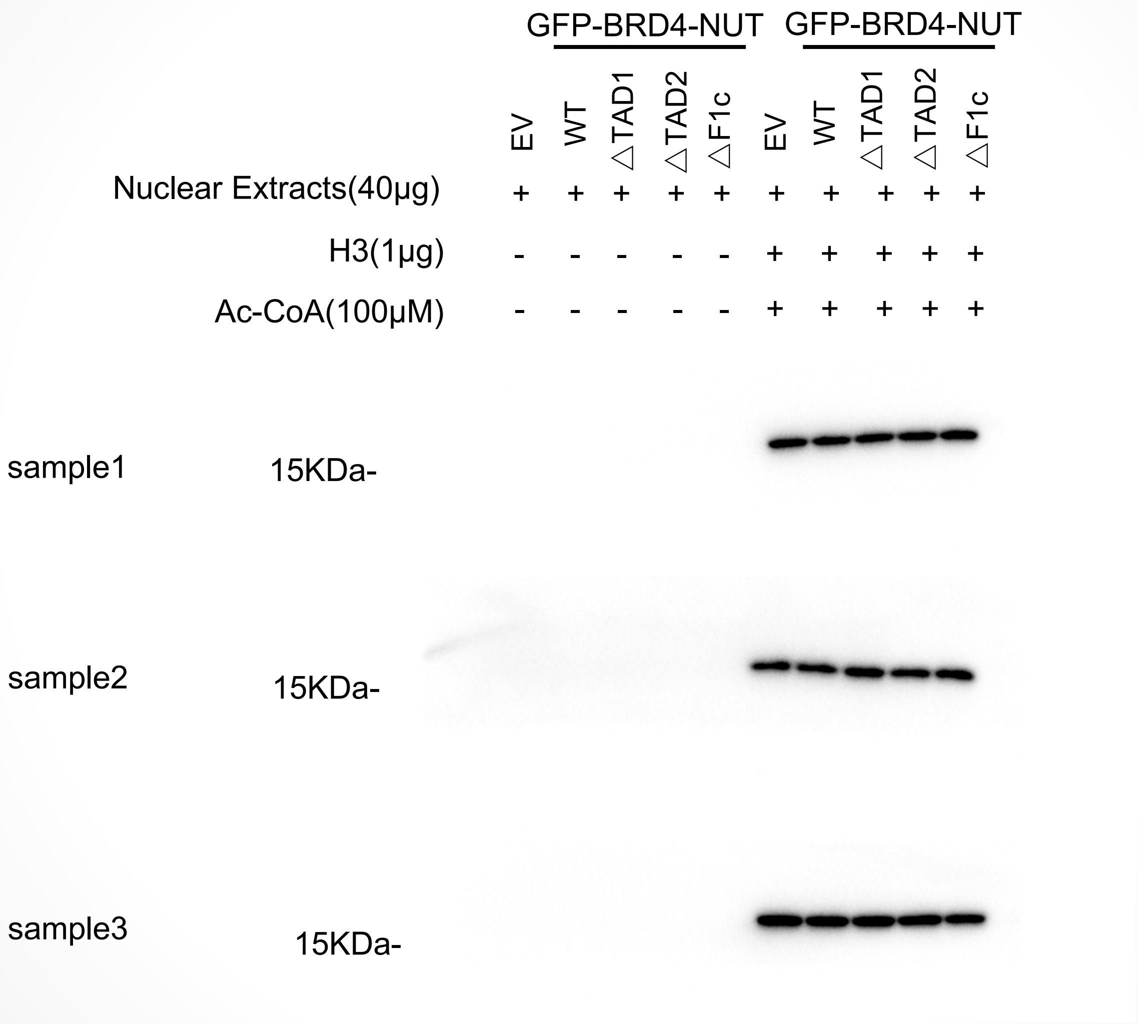
GFP-BRD4-NUT
GFP-BRD4-NUT
EV
WT
△TAD1
△TAD2
△F1c
EV
WT
△TAD1
△TAD2
△F1c
Nuclear Extracts(40μg) + + + + + + + + + +
H3(1μg) - - - - - + + + + +
Ac-CoA(100μM) - - - - - + + + + +
sample1
15KDa-
sample2
15KDa-
sample3
15KDa-

Figure 5b

H3K27ac

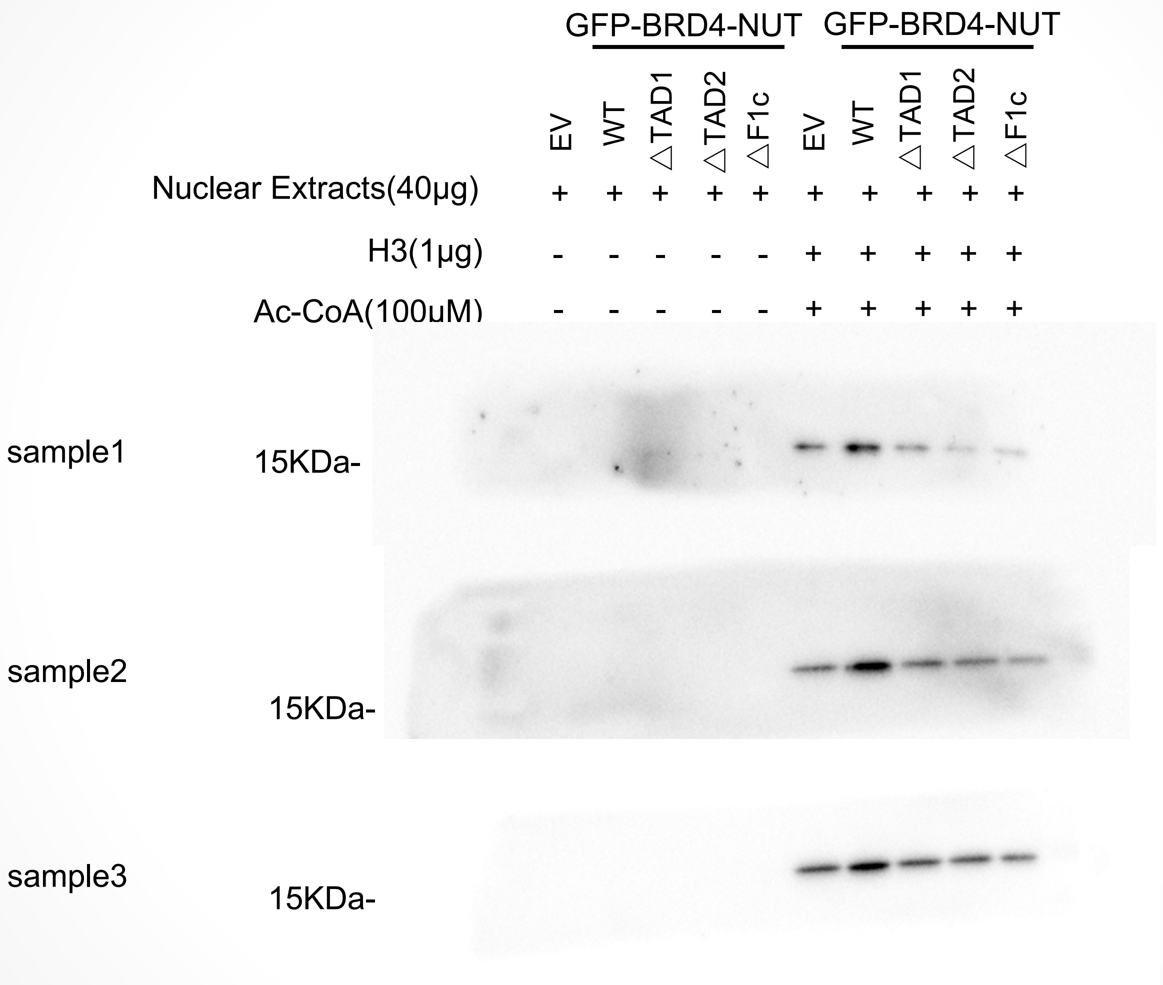

H3K27ac	1.00	1.98	0.63	0.45	0.34
	1.00	2.16	1.06	0.90	0.53
	1.00	1.62	1.03	0.95	0.75
Ave	1.00	1.92	0.90	0.77	0.54
P value		0.0043	0.0082	0.0067	0.0022

Figure 5b

H3K18ac

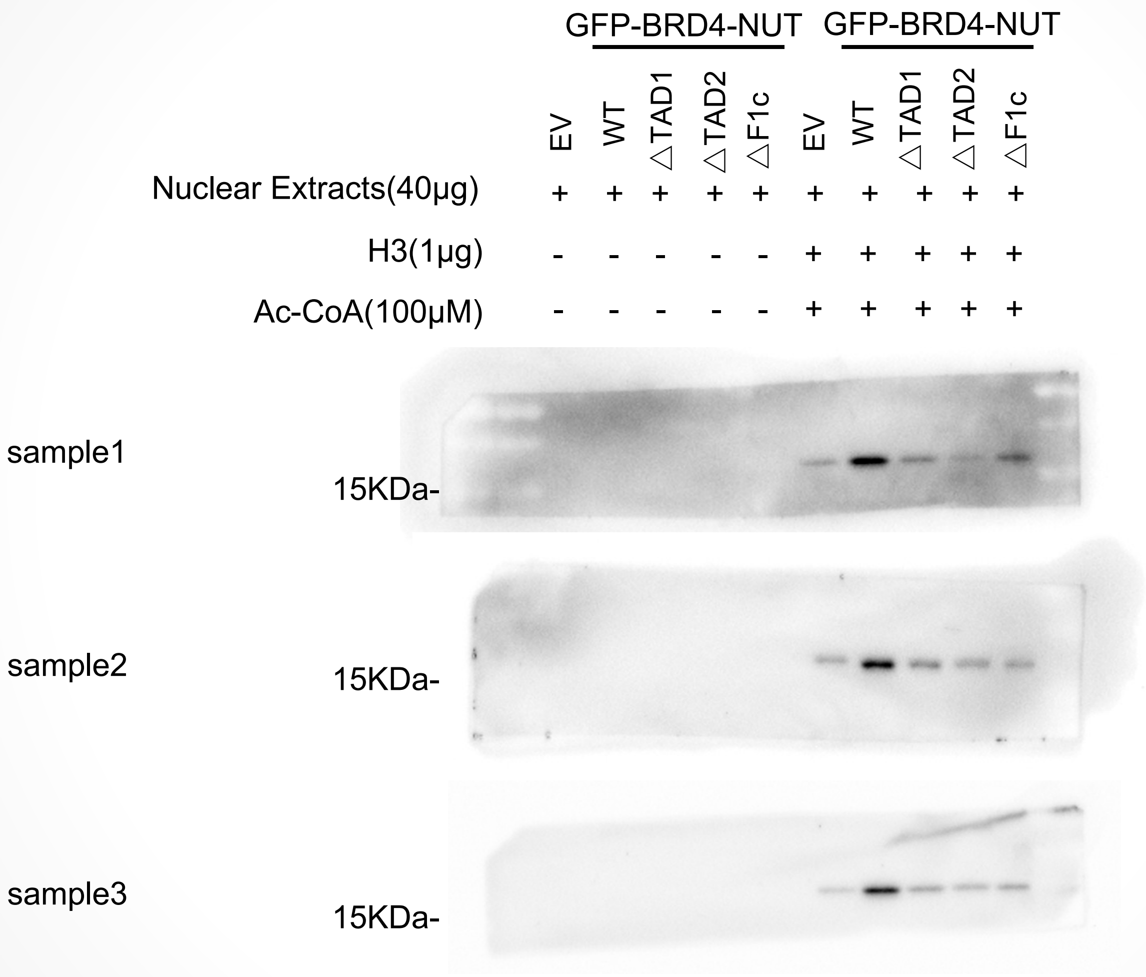

H3K18ac	1.00	5.75	1.46	1.08	2.25
	1.00	3.39	1.40	1.07	1.05
	1.00	5.93	2.07	1.56	2.01
Ave	1.00	5.02	1.64	1.23	1.77
P value		0.0079	0.0161	0.0104	0.0220

Figure 5b

H3K56ac

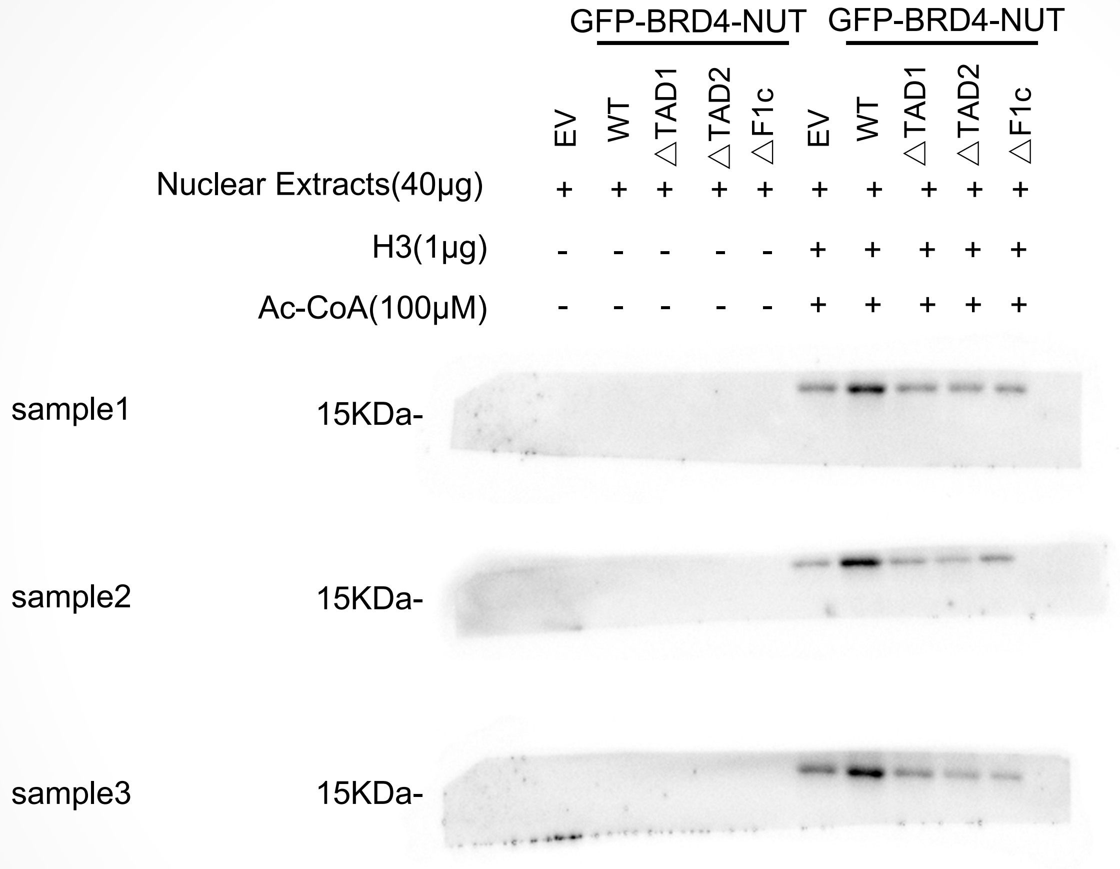

H3K56ac	1.00	2.28	0.97	0.81	0.79
	1.00	3.58	1.15	0.84	0.91
	1.00	1.90	0.62	0.48	0.43
Ave	1.00	2.59	0.91	0.71	0.71
P value		0.0353	0.0344	0.0227	0.0237

Figure 7b

Fig 6B-Alx1

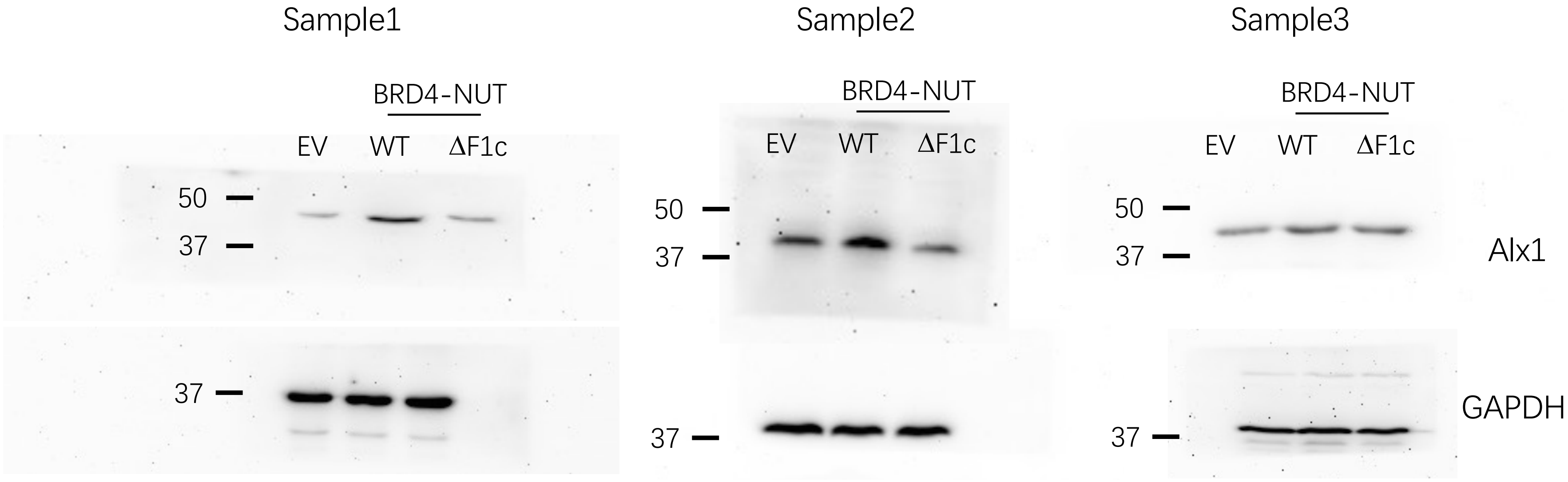

Alx1	EV	WT	△F1c
1	1.00	2.09	1.19
2	1.00	1.44	0.92
3	1.00	1.59	1.06
Ave	1.00	1.70	1.06
P value	NA	0.0234	0.0385

Figure 7b

Fig 6B-Snail

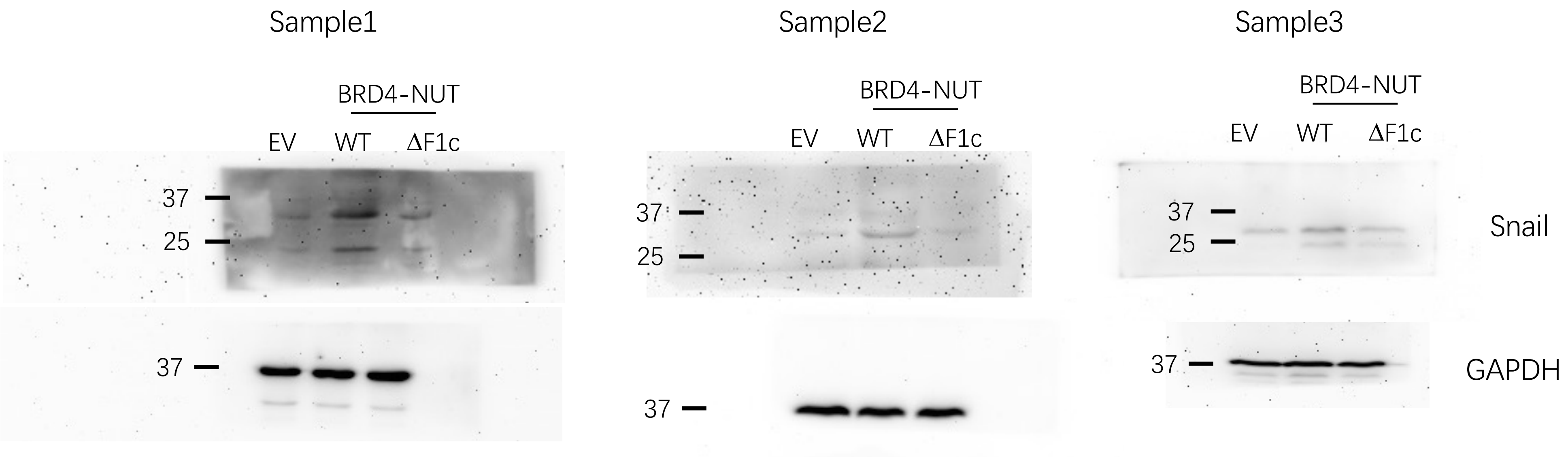

Snail	EV	WT	△F1c
**1**	1.00	3.36	1.11
**2**	1.00	2.20	0.66
**3**	1.00	1.73	0.67
**Ave**	1.00	2.43	0.82
**P value**	NA	0.0420	0.0334

Figure 7b

Fig 6B-E-Cad

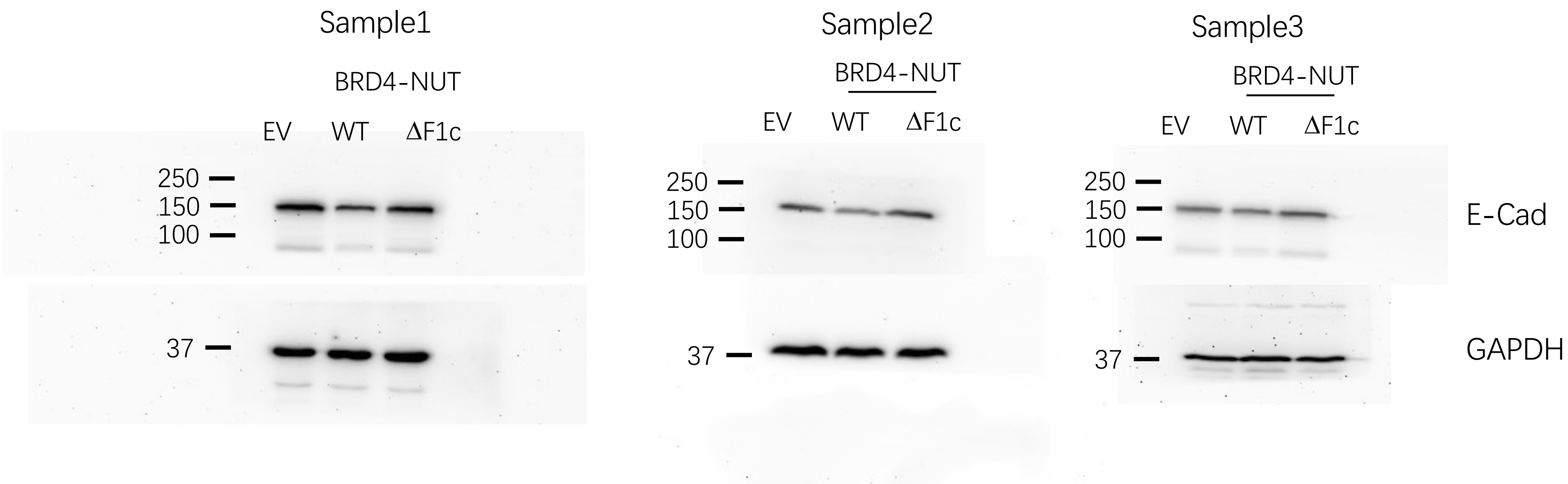

E-cad	EV	WT	△F1c
1	1.00	0.52	0.90
2	1.00	0.68	0.96
3	1.00	0.78	1.18
Ave	1.00	0.66	1.01
P value	NA	0.0116	0.0361

Figure 7b

Fig 6B-Vimentin

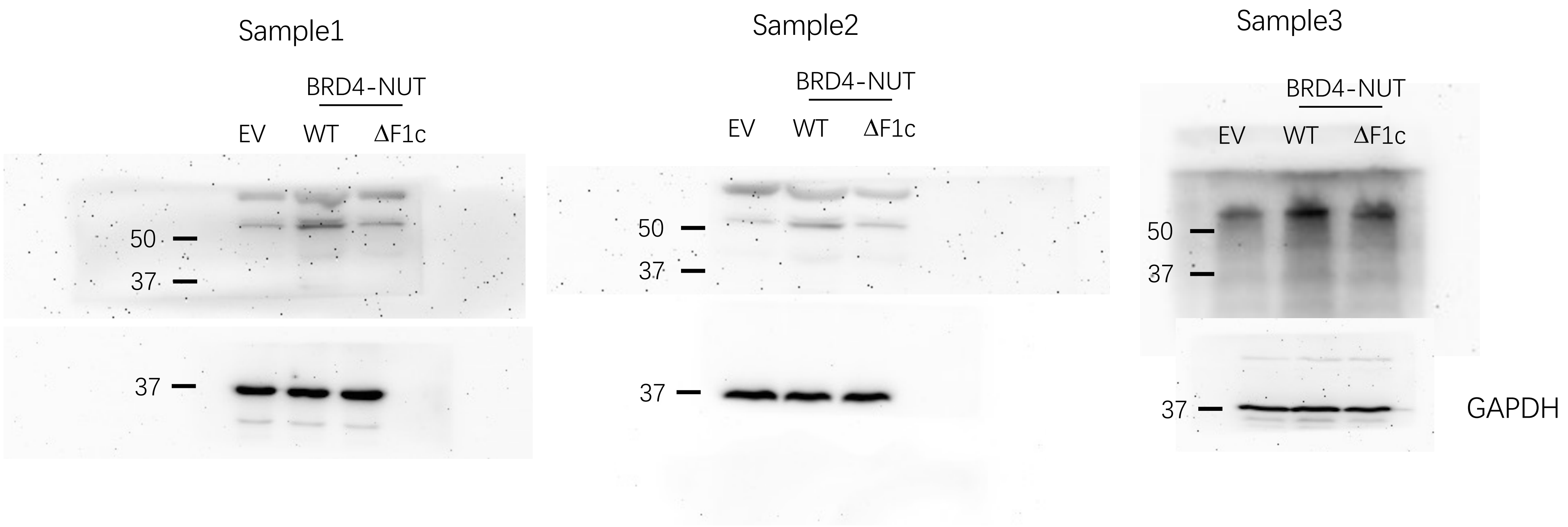

Vimentin	EV	WT	△F1c
**1**	1.00	2.52	1.24
**2**	1.00	2.05	1.07
**3**	1.00	3.01	1.75
**Ave**	1.00	2.53	1.35
**P value**	NA	0.0054	0.0272

Figure 7b

Fig 6B-N-Cad

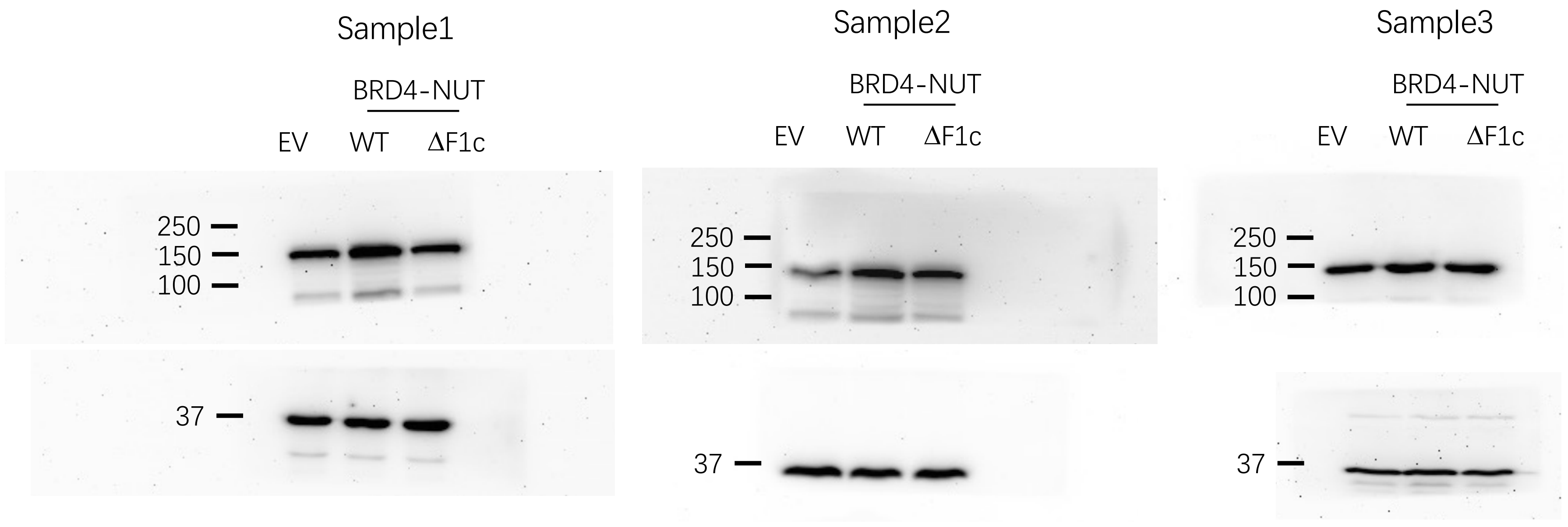

N-Cad	EV	WT	△F1c
**1**	1.00	1.52	0.95
**2**	1.00	1.71	1.30
**3**	1.00	1.29	0.84
**Ave**	1.00	1.51	1.03
**P value**	NA	0.0139	0.0611

Figure 7c

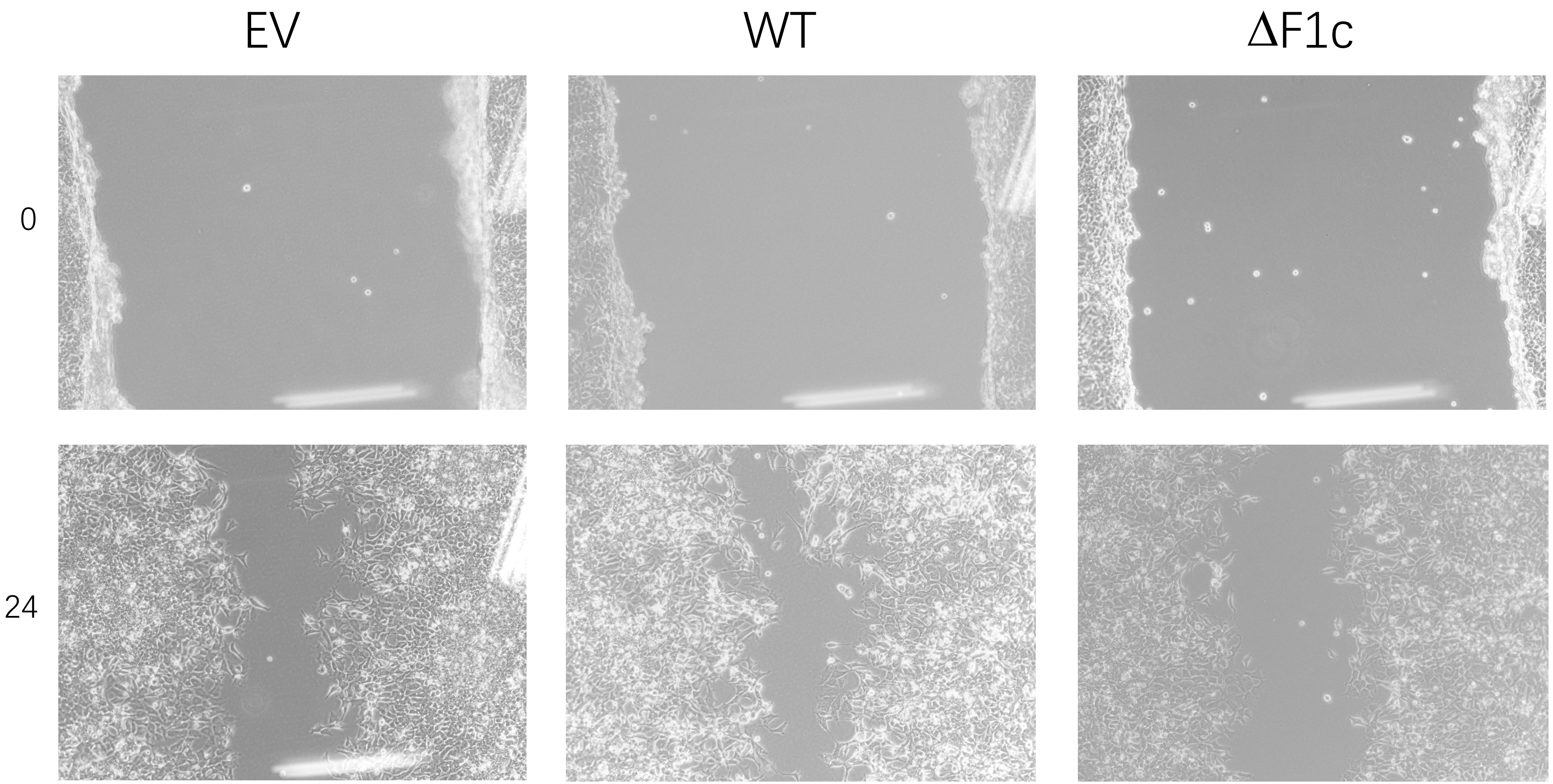

Figure 7e

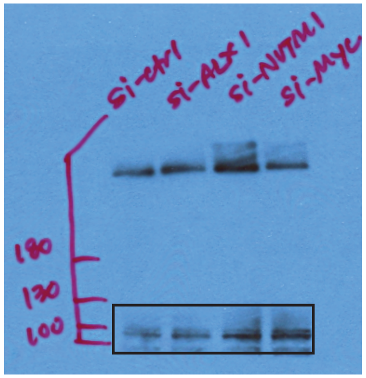

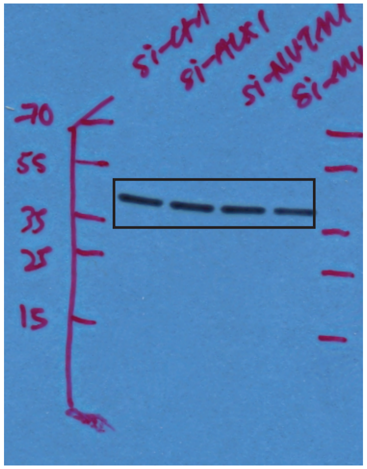

ED Figure 7a

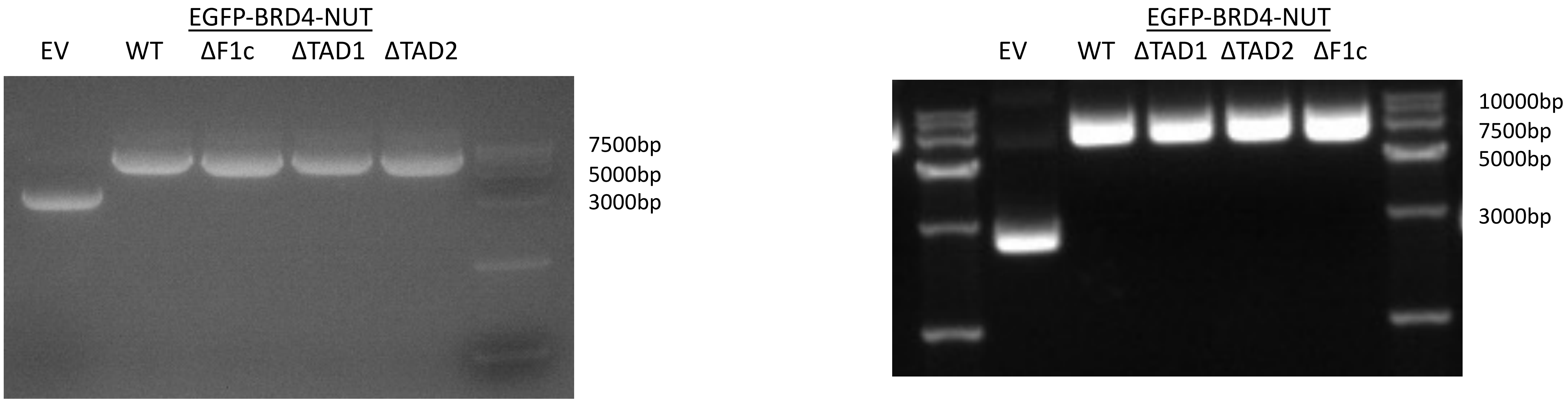
EGFP-BRD4-NUT
EV
WT
ΔF1c
ΔTAD1
ΔTAD2
7500bp
5000bp
3000bp
EGFP-BRD4-NUT
EV
WT
ΔTAD1
ΔTAD2
ΔF1c
10000bp
7500bp
5000bp
3000bp

ED-Figure 7c

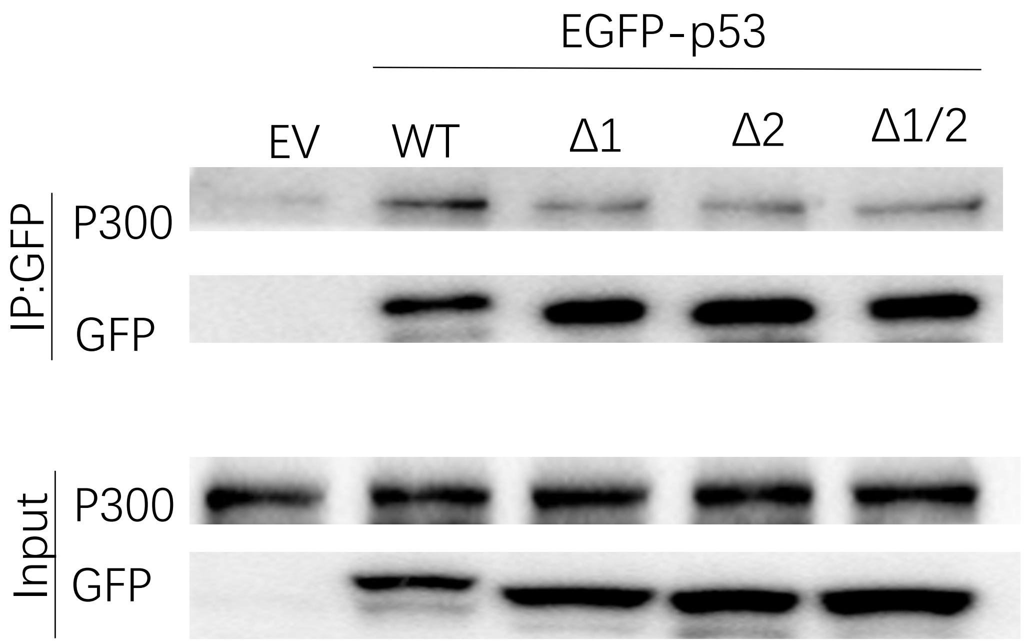

ED-Figure 7c

P300

IP

INPUT

220919
AFFINITY-5

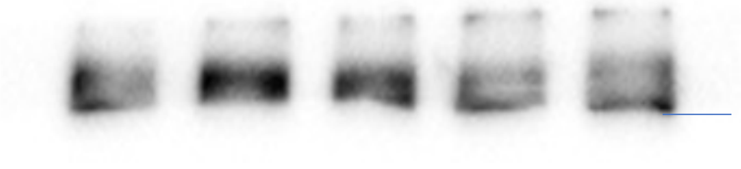

250kD

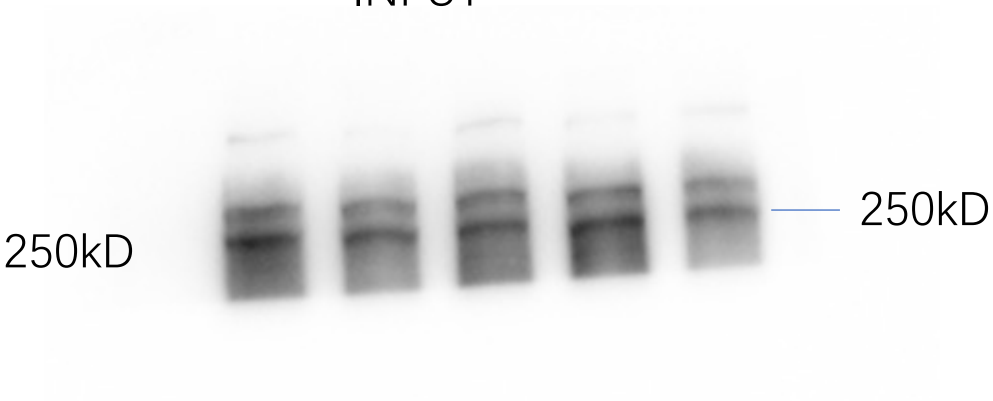

250kD

220825
AFFINITY-10

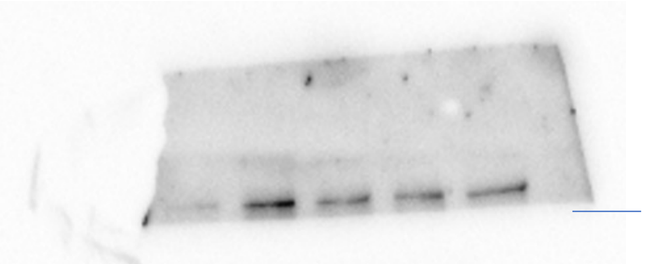

250kD

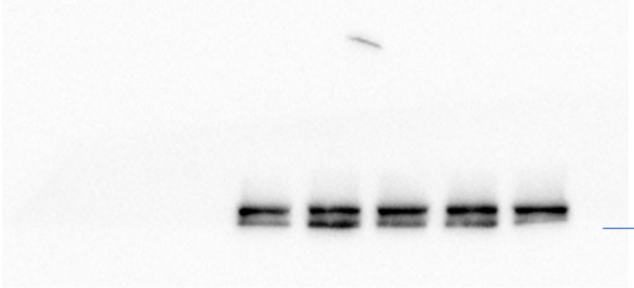

250kD

GFP

ED-Figure 7c

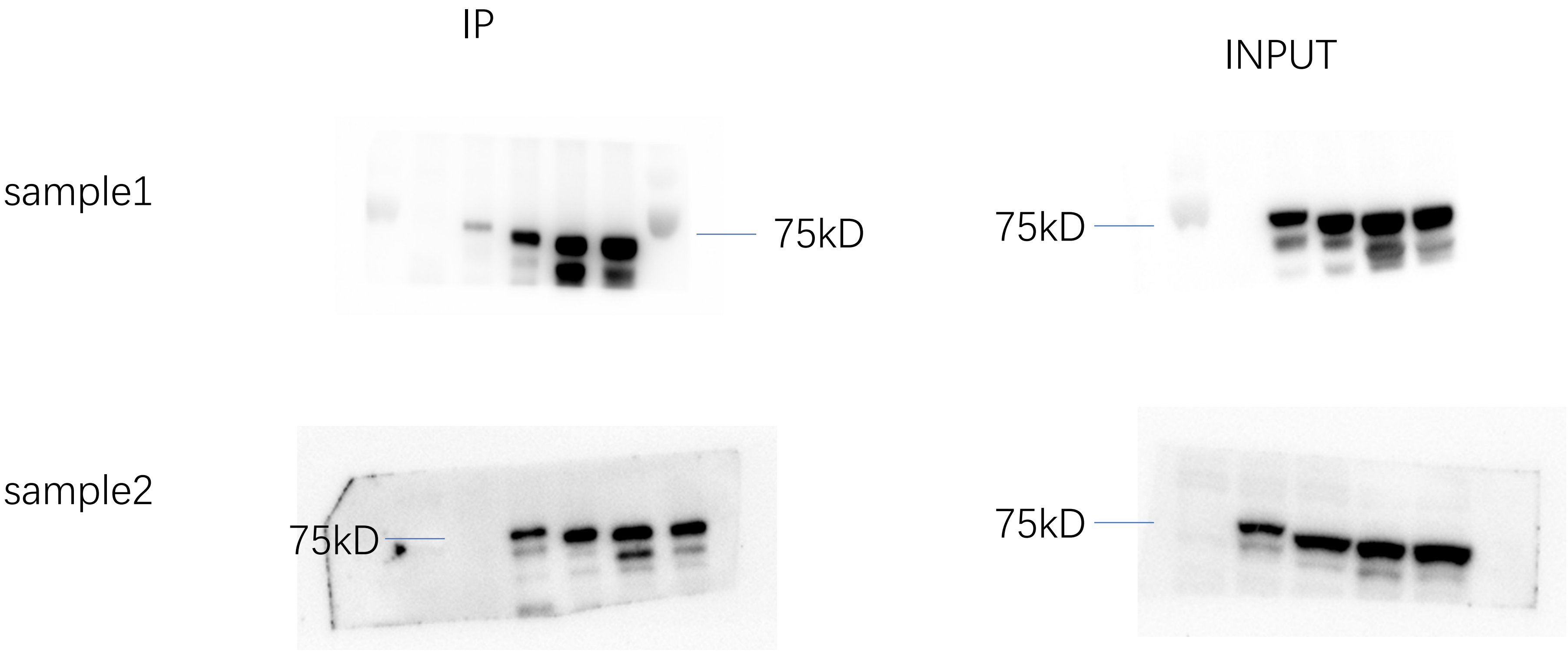

p300

ED-Figure 7d

Sample 1

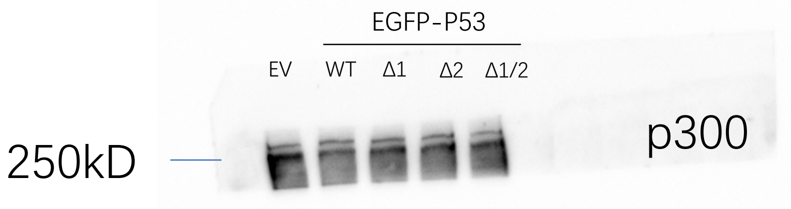

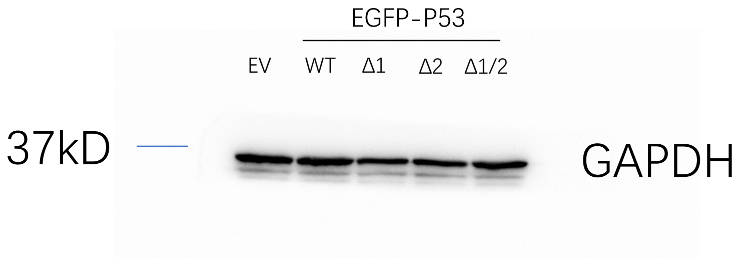

Sample 2

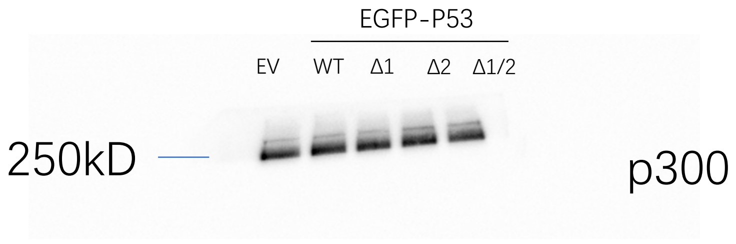

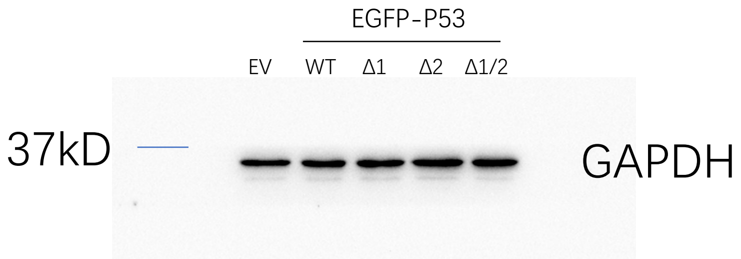

Sample 3

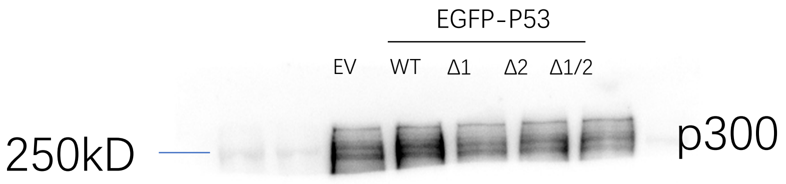

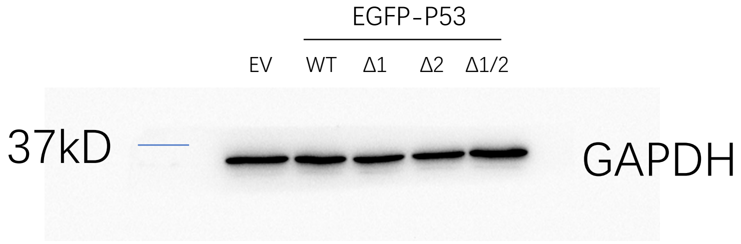

p300ac

ED-Figure 7d

Sample 1

Sample 2

Sample 3

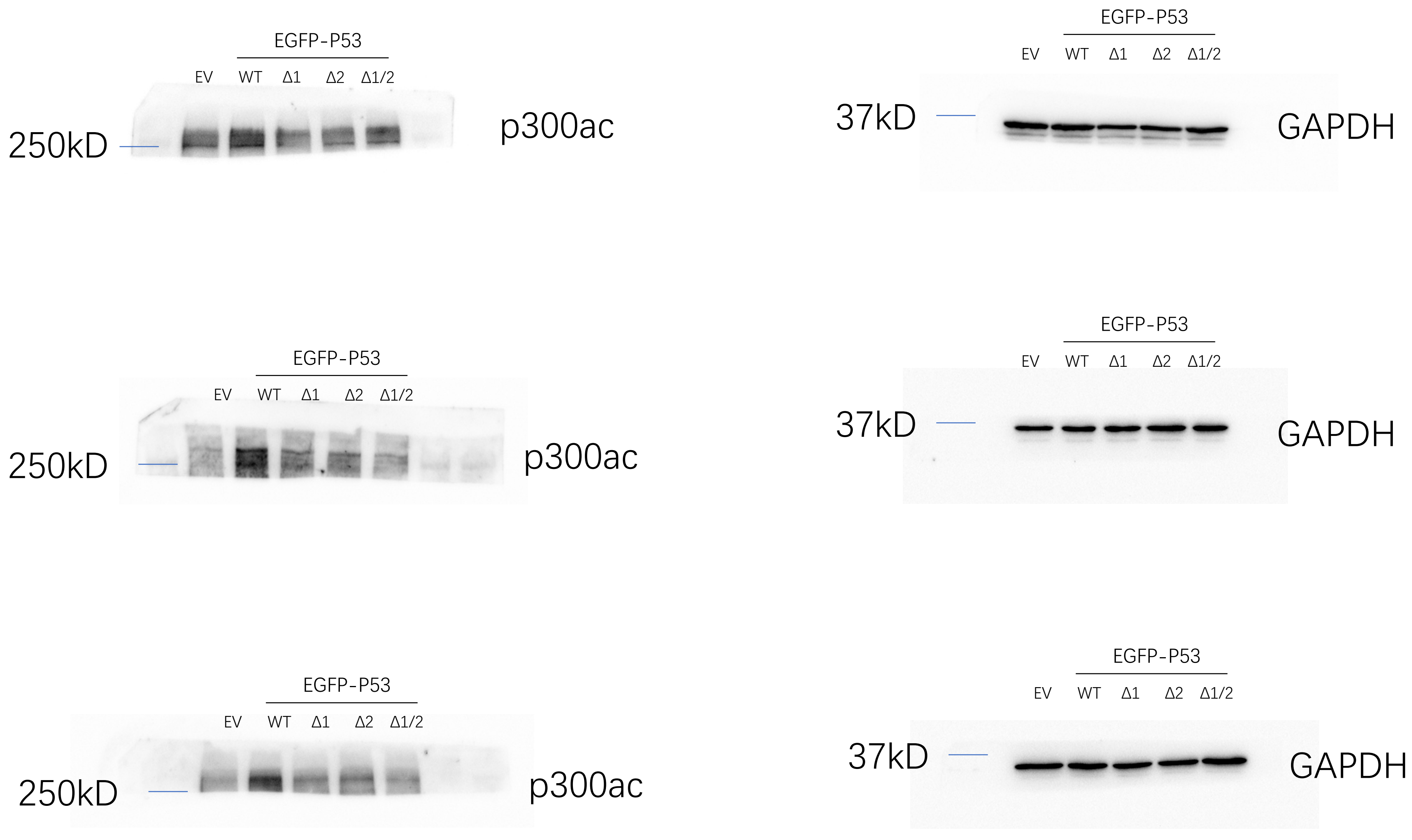

GFP

ED-Figure 7d

Sample 1

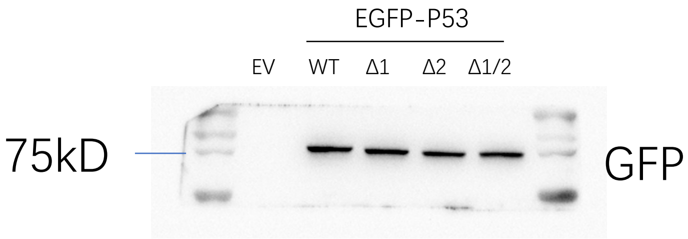

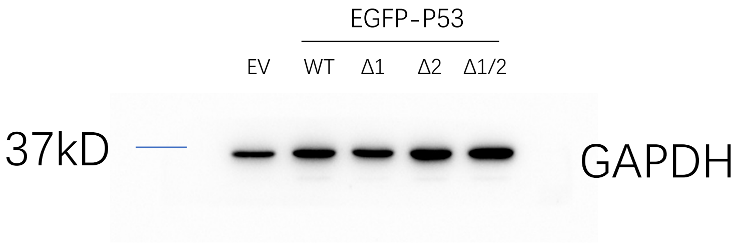

Sample 2

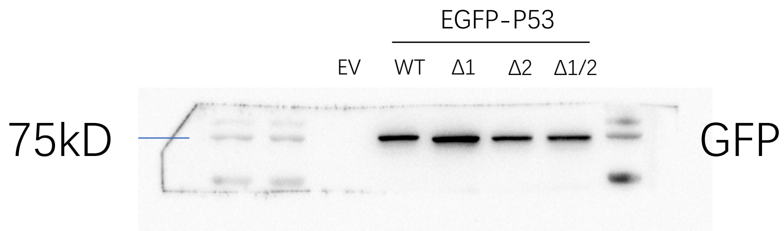

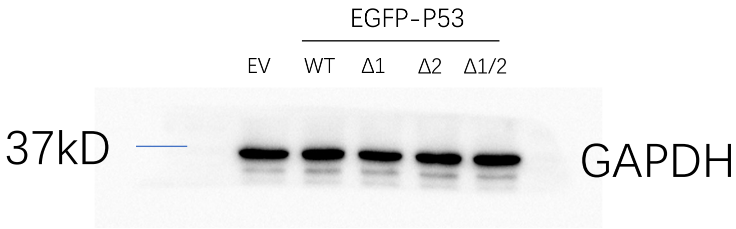

Sample 3

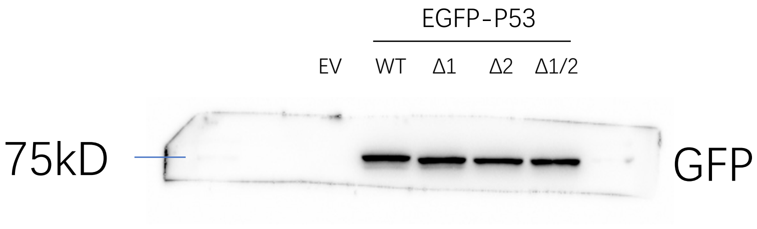

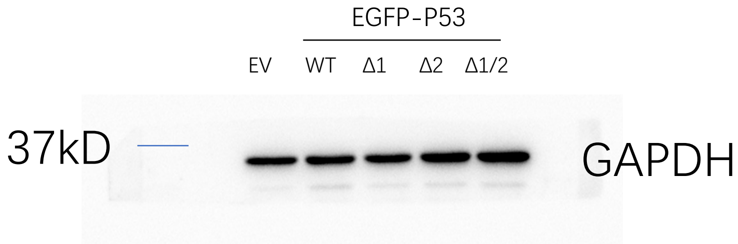

P53ac-80kD

ED-Figure 7d

Sample 1

EGFP-P53
EV WT Δ1 Δ2 Δ1/2

75kD 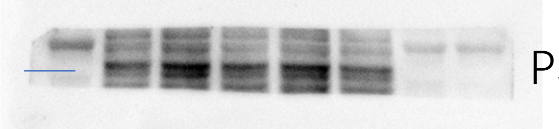 P53ac-80kD

EGFP-P53
EV WT Δ1 Δ2 Δ1/2

37kD 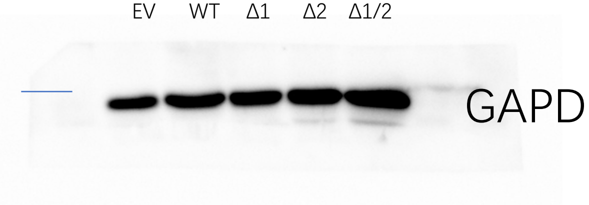 GAPDH

Sample 2

EGFP-P53
EV WT Δ1 Δ2 Δ1/2

75kD 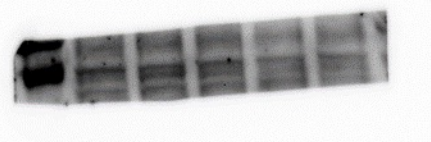 P53ac-80kD

EGFP-P53
EV WT Δ1 Δ2 Δ1/2

37kD 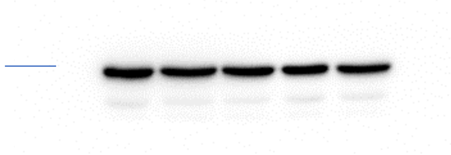 GAPDH

Sample 3

EGFP-P53
EV WT Δ1 Δ2 Δ1/2

75kD 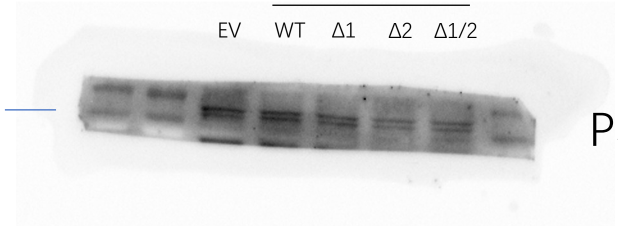 P53ac-80kD

EGFP-P53
EV WT Δ1 Δ2 Δ1/2

37kD 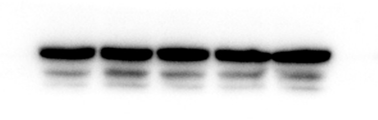 GAPDH

P53ac-53kD

ED-Figure 7d

Sample 1

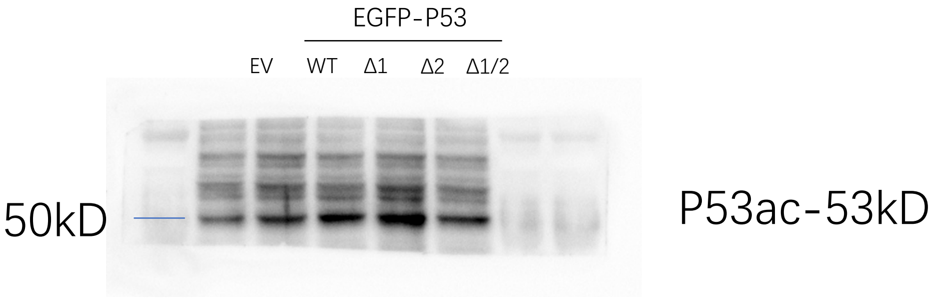

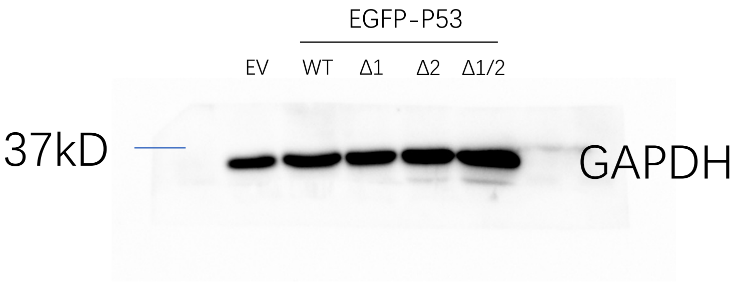

Sample 2

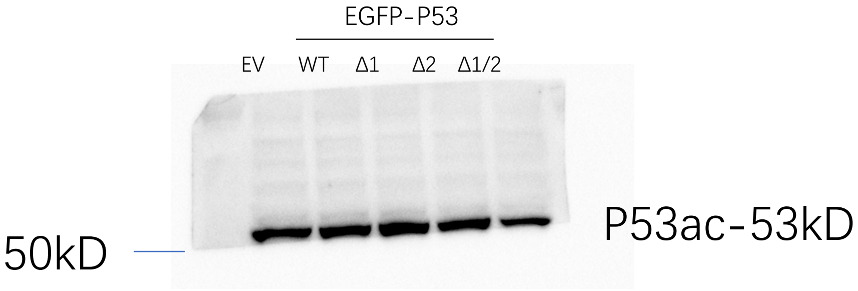

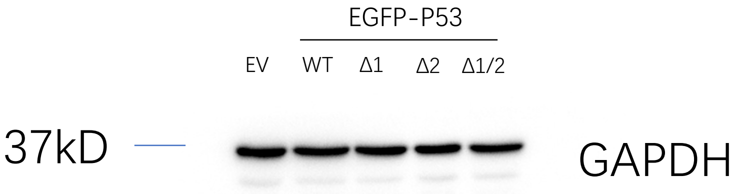

Sample 3

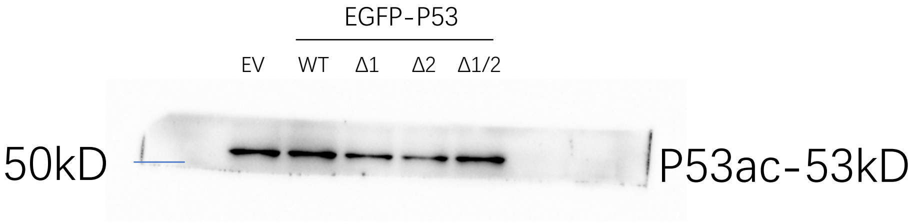

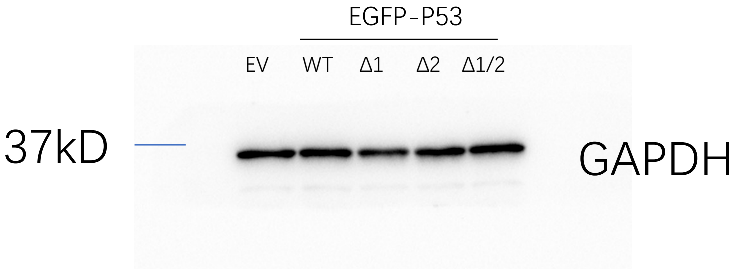

H3K18ac

ED-Figure 7d

Sample 1

Sample 2

Sample 3

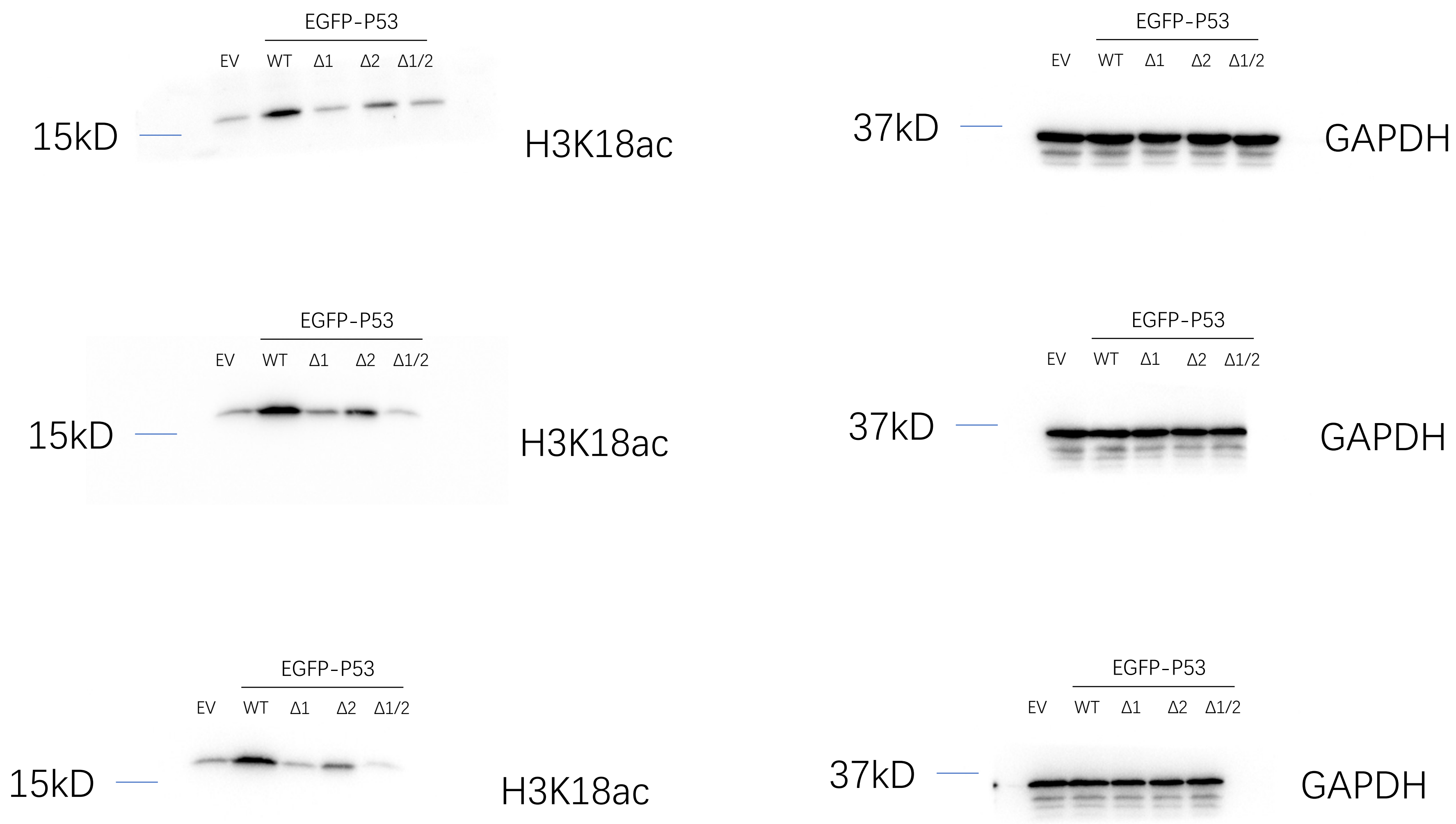

H3K27ac

ED-Figure 7d

Sample 1

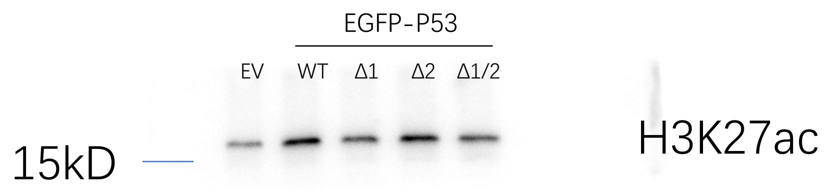

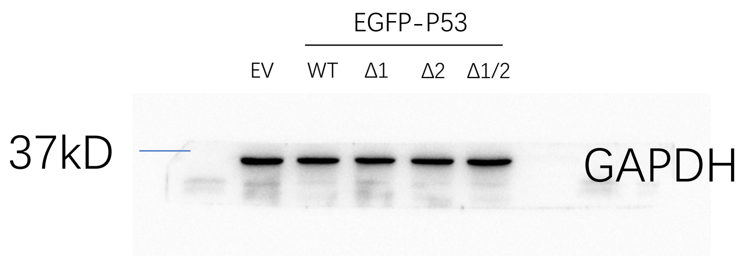

Sample 2

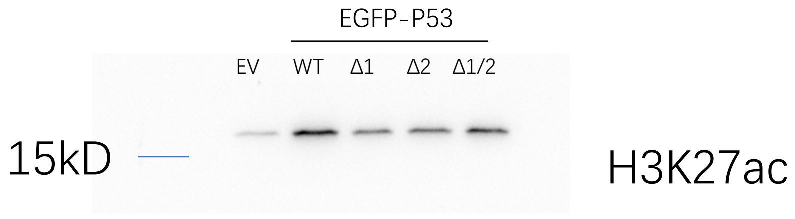

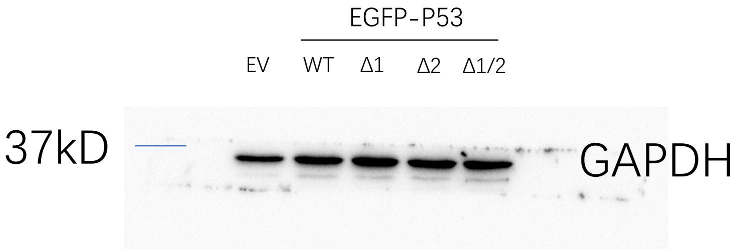

Sample 3

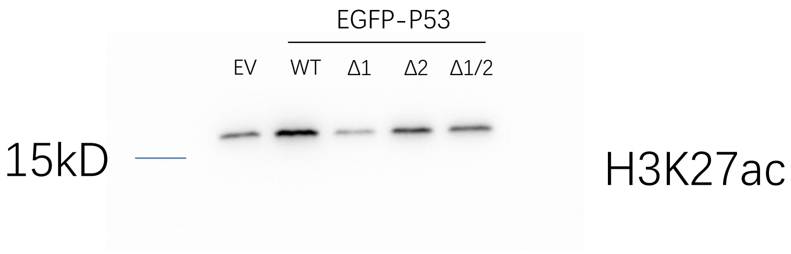

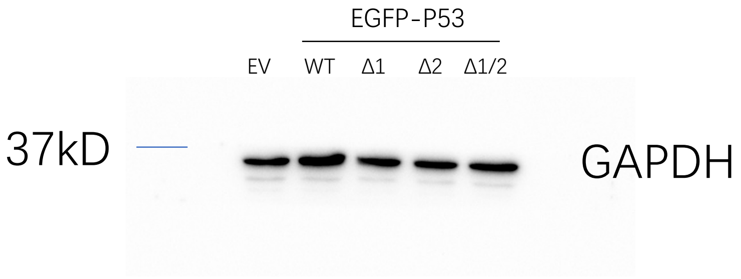

H3K56ac

ED-Figure 7d

Sample 1

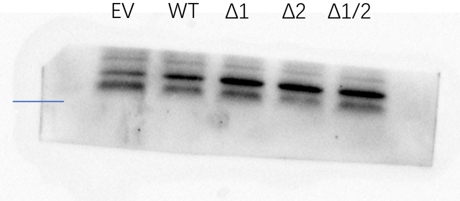

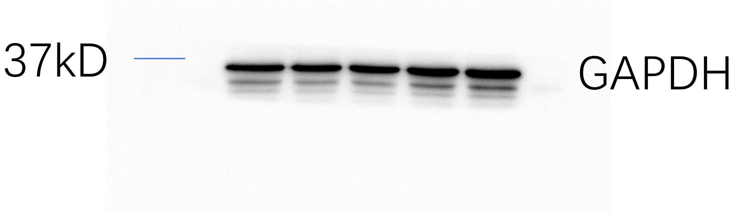

Sample 2

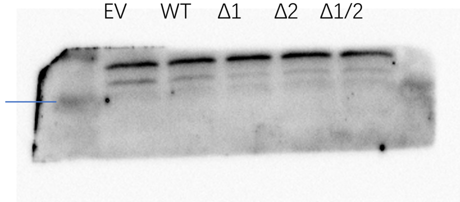

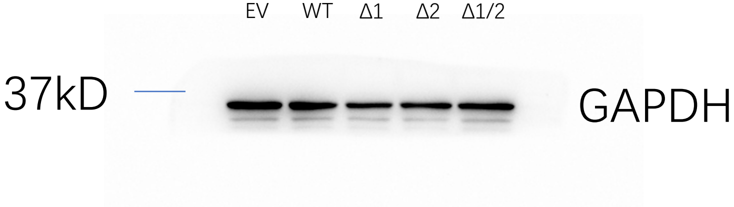

Sample 3

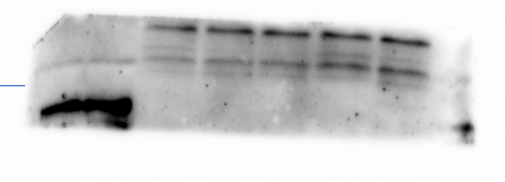

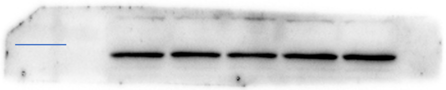

H4K12ac

ED-Figure 7d

Sample 1

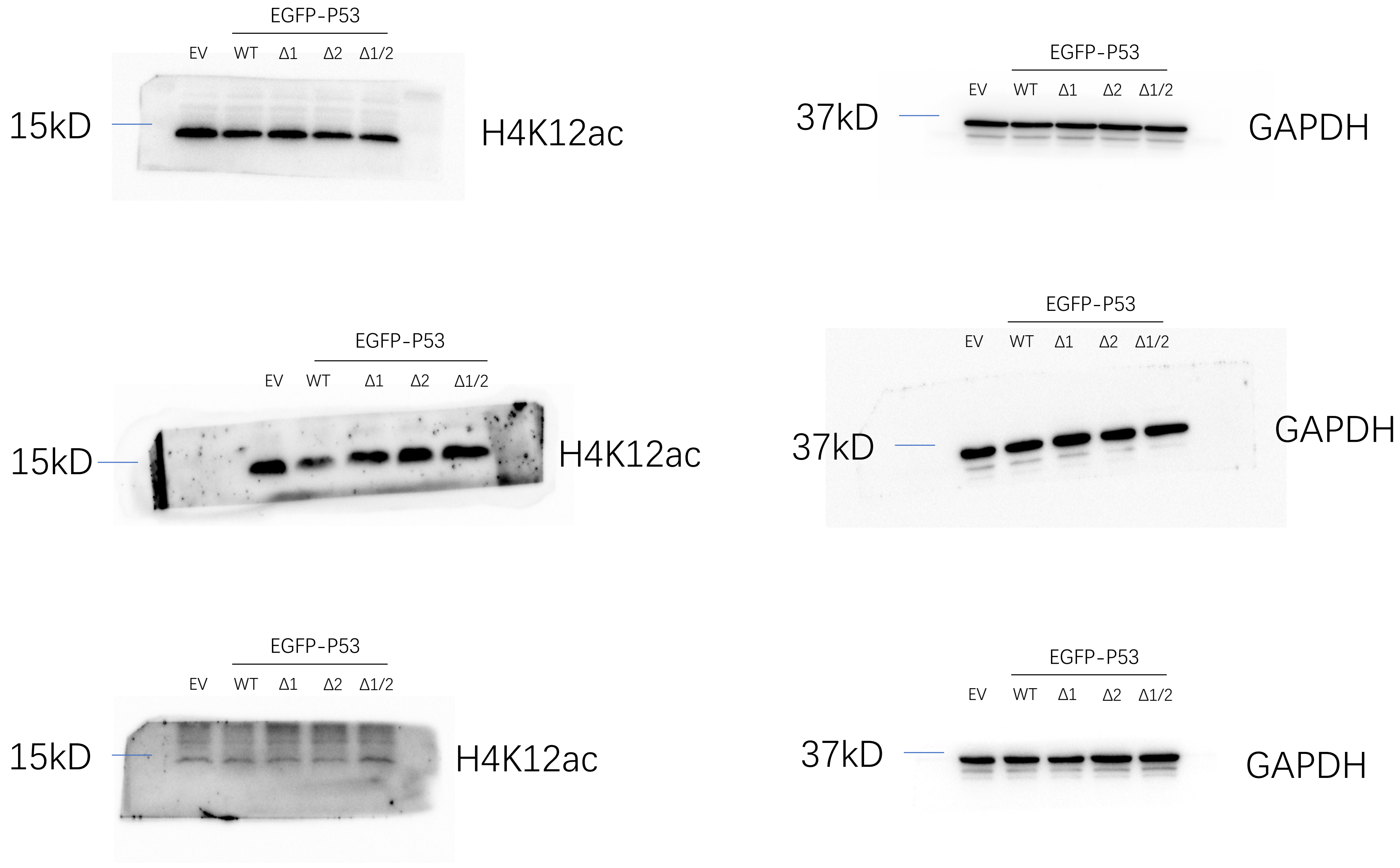

p300

ED-Figure 7e

Sample 1

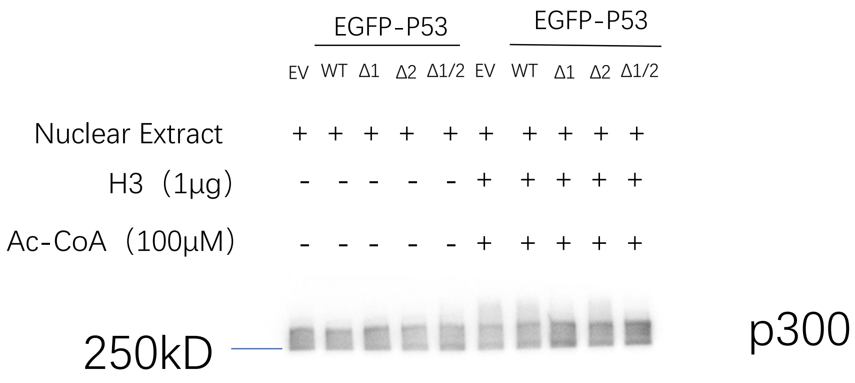

Sample 2

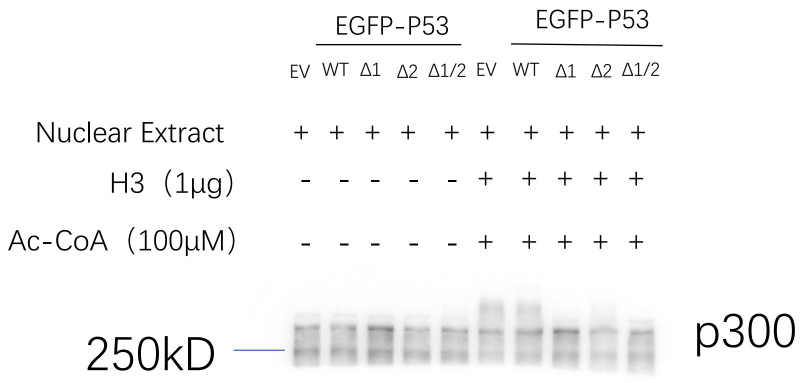

Sample 3

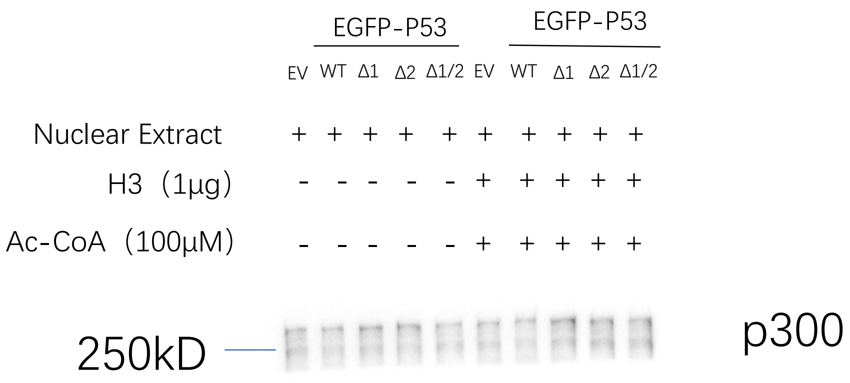

p300ac

ED-Figure 7e

Sample 1

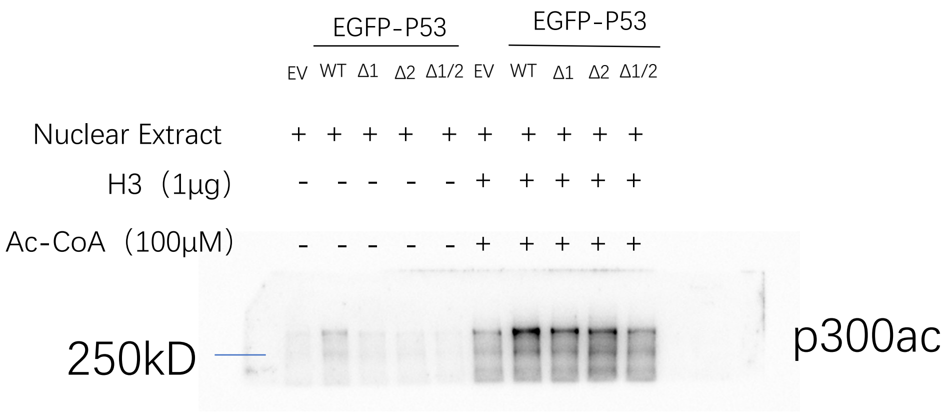

Sample 2

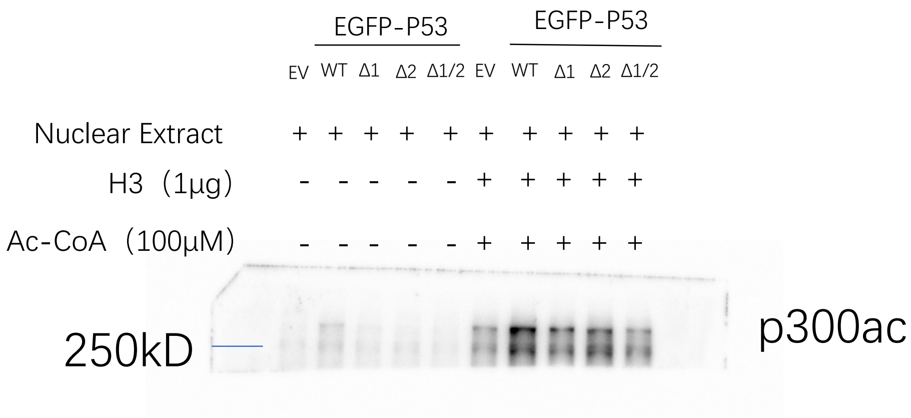

Sample 3

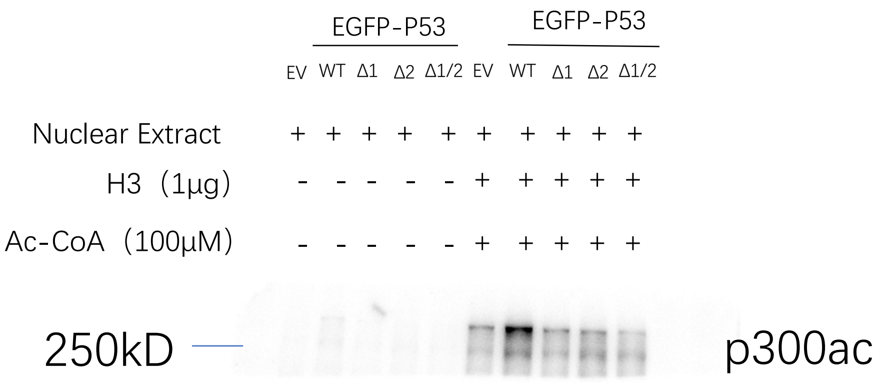

GFP

ED-Figure 7e

Sample 1

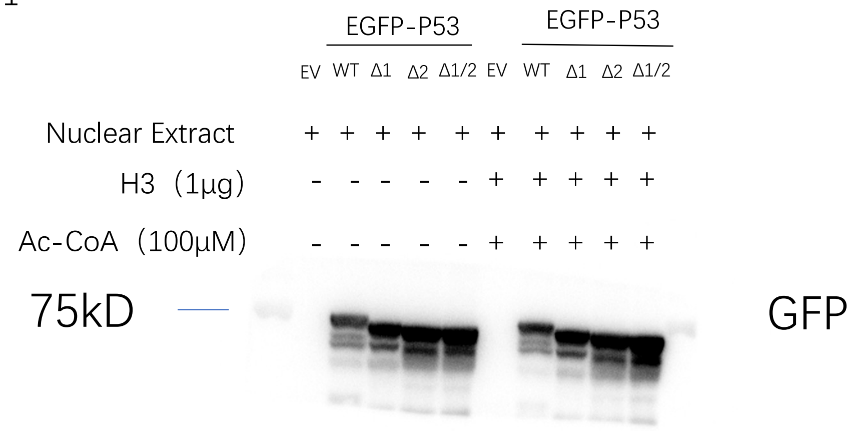

Sample 2

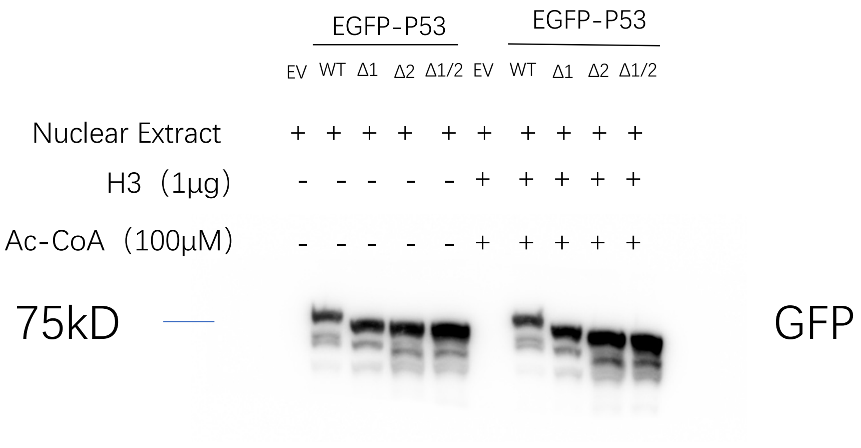

Sample 3

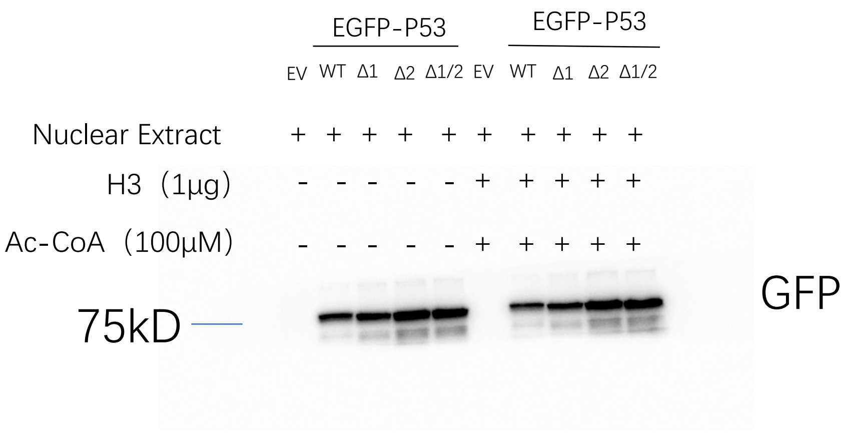

P53ac-80KD

ED-Figure 7e

Sample 1

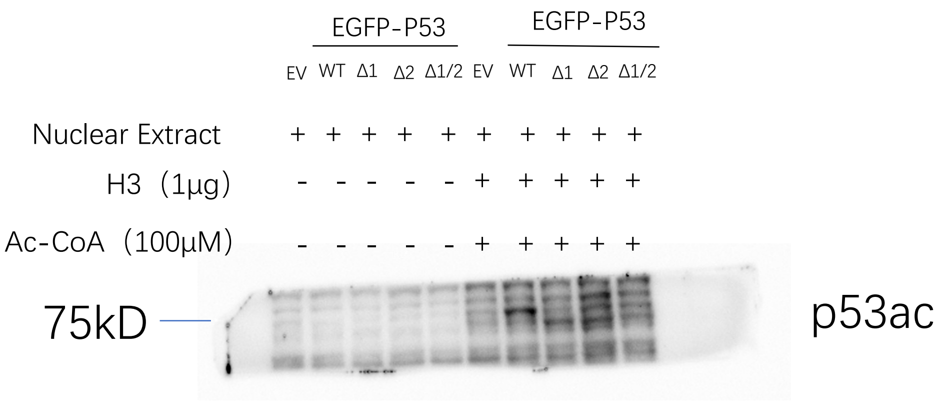

Sample 2

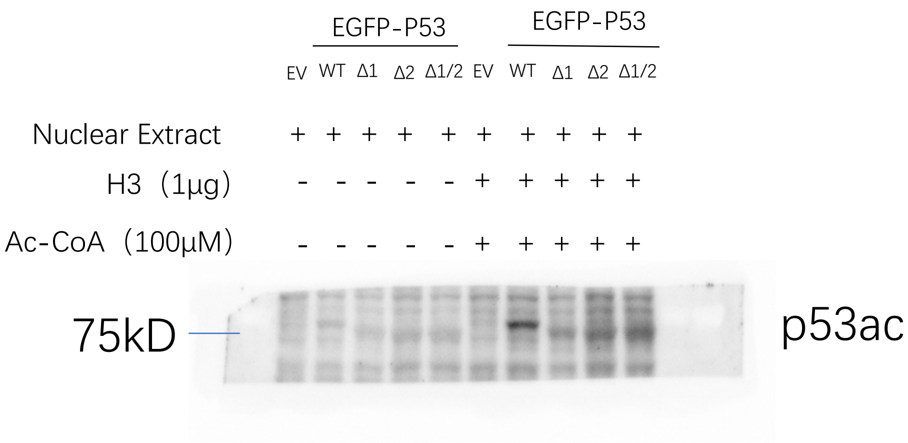

Sample 3

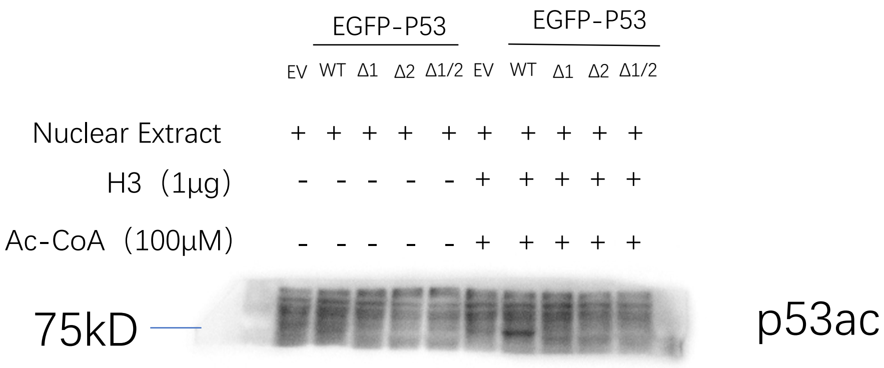

P53ac-53KD

ED-Figure 7e

Sample 1

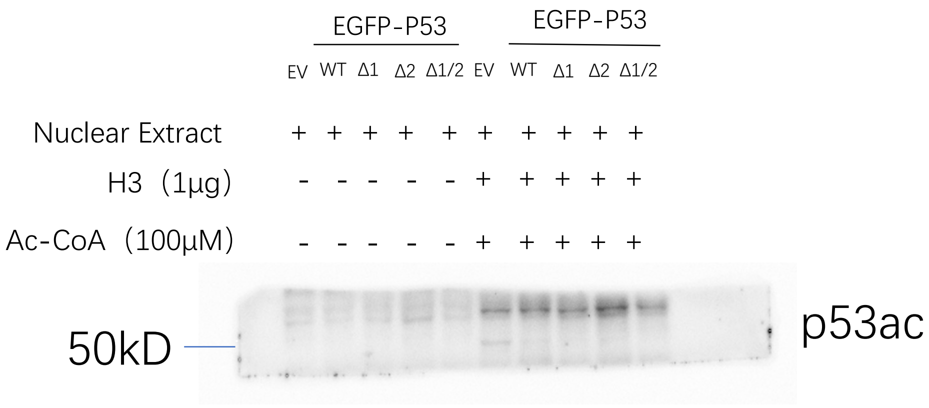

Sample 2

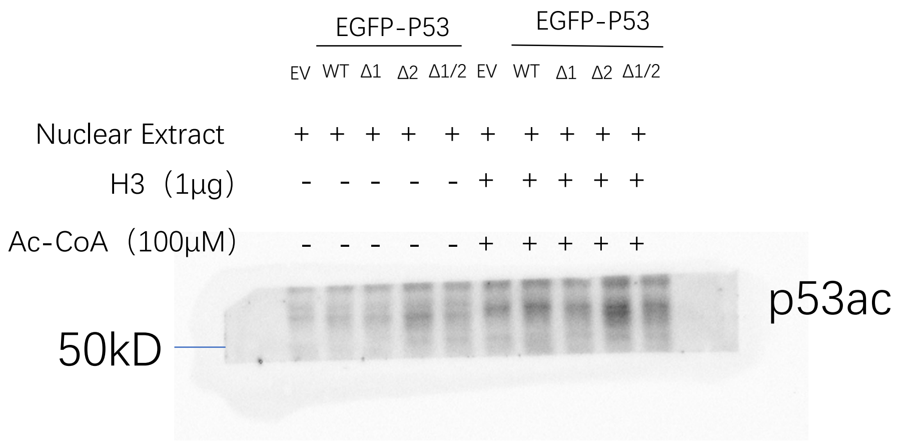

Sample 3

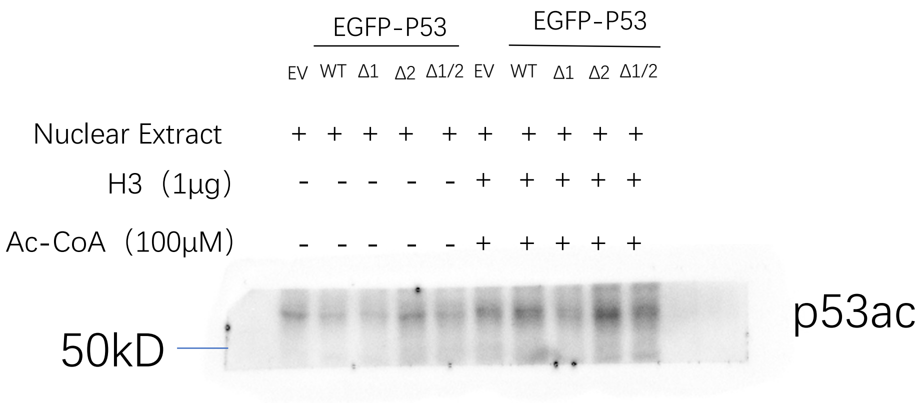

H3K18ac

ED-Figure 7e

Sample 1

	EGFP-P53					EGFP-P53				
	EV	WT	Δ1	Δ2	Δ1/2	EV	WT	Δ1	Δ2	Δ1/2
Nuclear Extract	+	+	+	+	+	+	+	+	+	+
H3 (1μg)	-	-	-	-	-	+	+	+	+	+
Ac-CoA (100μM)	-	-	-	-	-	+	+	+	+	+

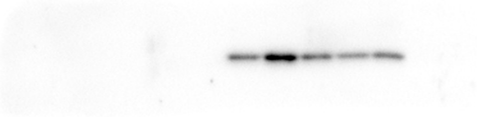

Sample 2

	EGFP-P53					EGFP-P53				
	EV	WT	Δ1	Δ2	Δ1/2	EV	WT	Δ1	Δ2	Δ1/2
Nuclear Extract	+	+	+	+	+	+	+	+	+	+
H3 (1μg)	-	-	-	-	-	+	+	+	+	+
Ac-CoA (100μM)	-	-	-	-	-	+	+	+	+	+

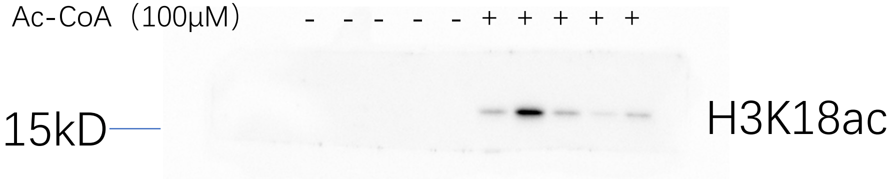

Sample 3

	EGFP-P53					EGFP-P53				
	EV	WT	Δ1	Δ2	Δ1/2	EV	WT	Δ1	Δ2	Δ1/2
Nuclear Extract	+	+	+	+	+	+	+	+	+	+
H3 (1μg)	-	-	-	-	-	+	+	+	+	+
Ac-CoA (100μM)	-	-	-	-	-	+	+	+	+	+

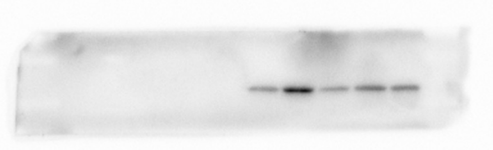

H3K27ac

ED-Figure 7e

Sample 1

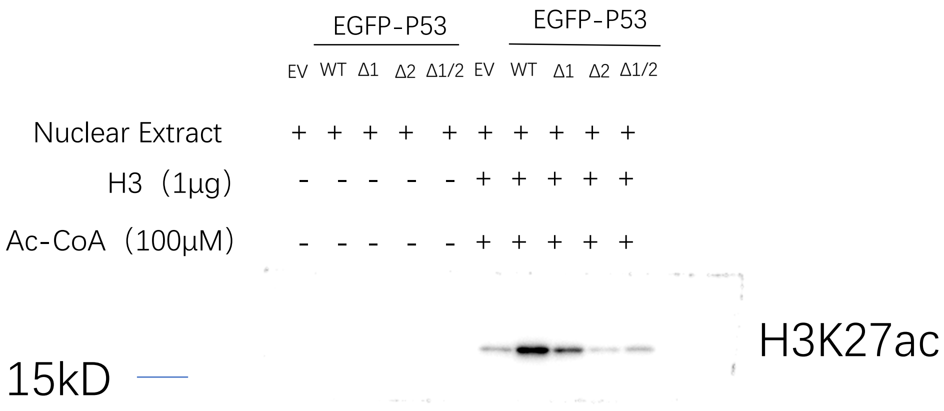

Sample 2

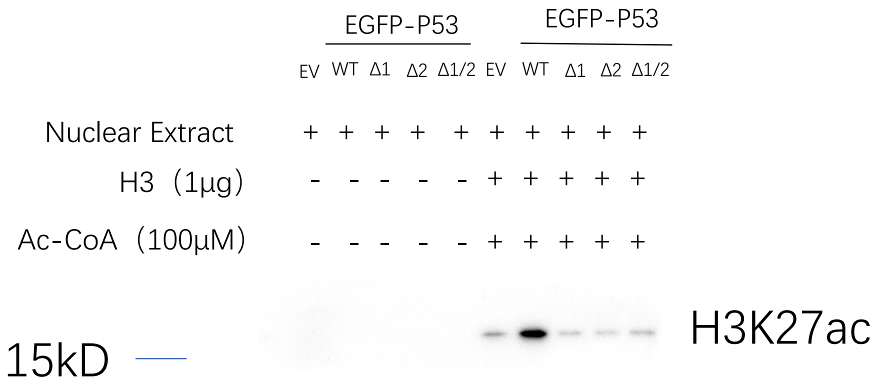

Sample 3

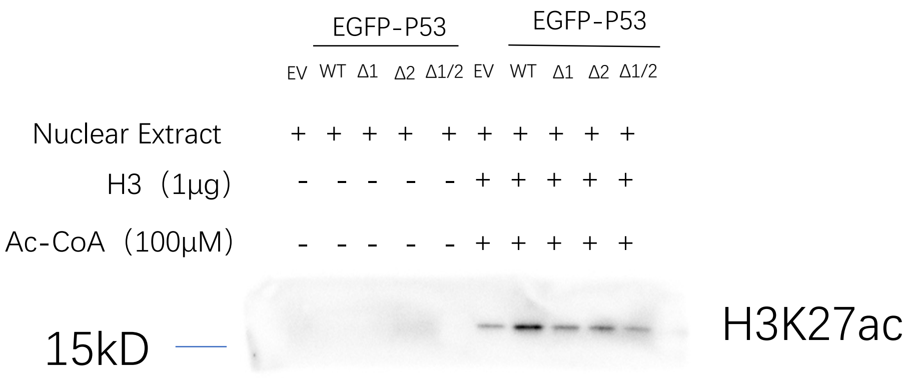

H3K56ac

ED-Figure 7e

Sample 1

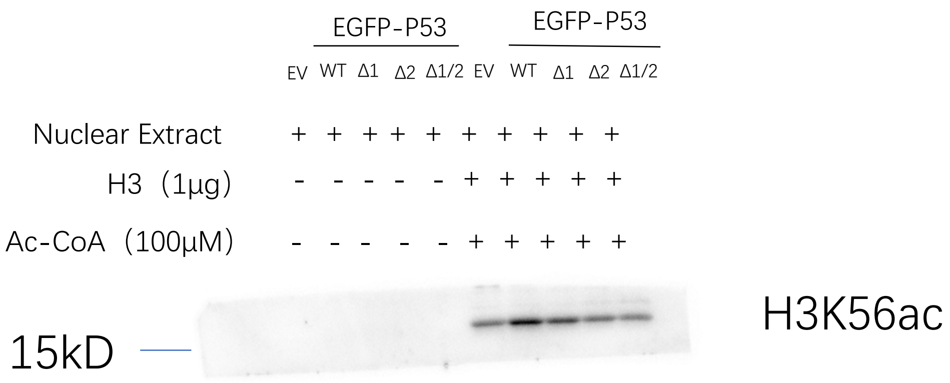

Sample 2

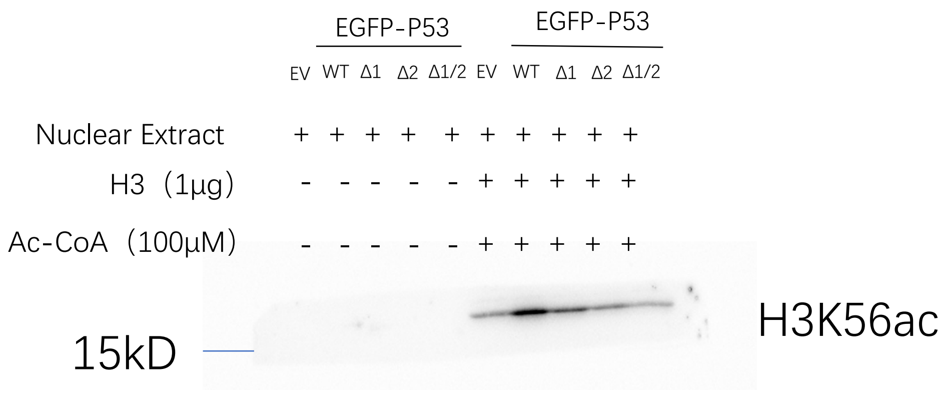

Sample 3

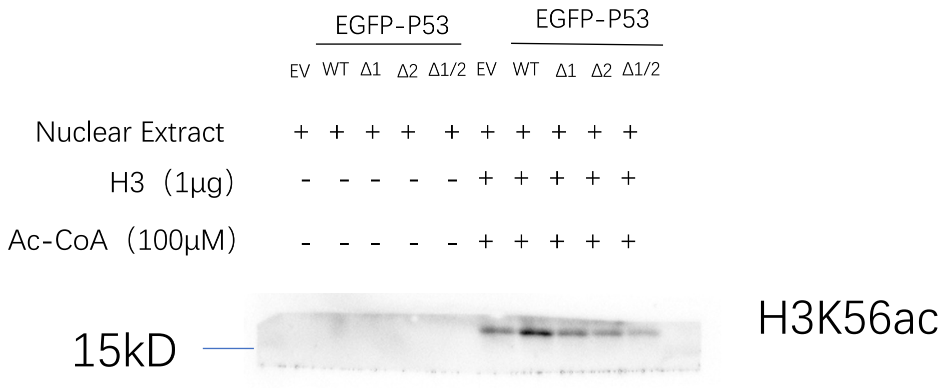

H3

ED-Figure 7e

Sample 1

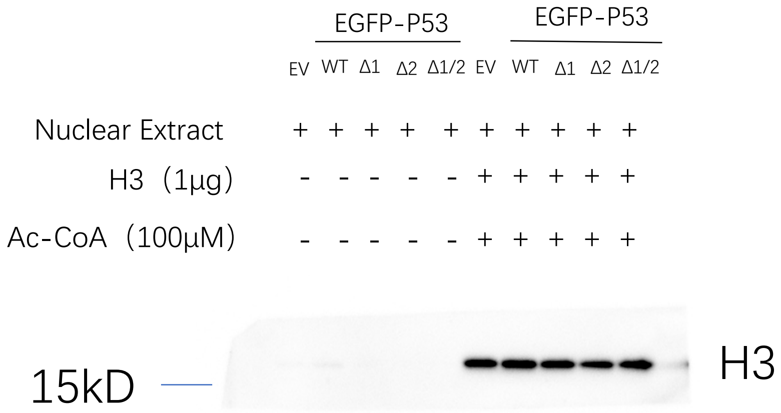

Sample 2

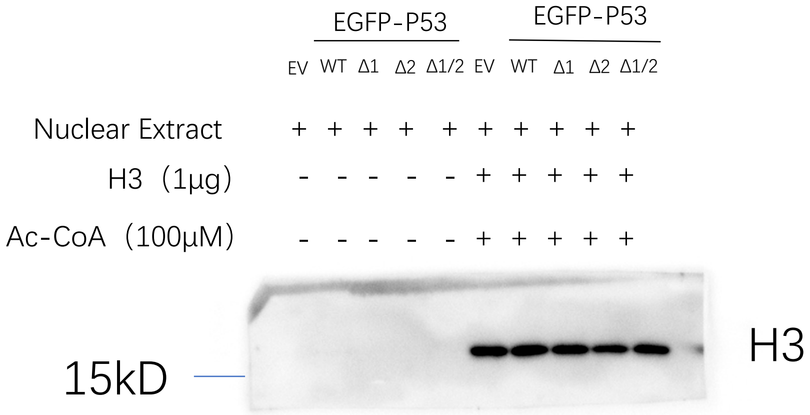

Sample 3

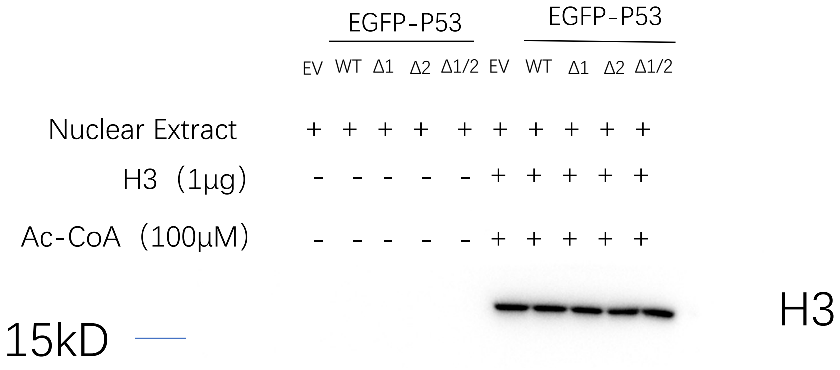

Supplement: Supplementary file 4 — Source Data [file 41467_2023_36063_MOESM4_ESM.zip › Source Data/202_Figures-Raw-data_NCOMMS-22-25872A_20221225_FINAL.pdf]
